# Engineering a 660 nm‐Responsive Optogenetic Inducer of Pyroptosis for Precision Cancer Therapy

**DOI:** 10.1002/advs.76768

**Published:** 2026-07-31

**Authors:** Mengkai Zhang, Zhichao Li, Yonghao Ma, Yangyang Sun, Wei Chen, Weiren Huang

**Affiliations:** ^1^ Medical Innovation Technology Transformation Center Shenzhen Second People's Hospital The First Affiliated Hospital of Shenzhen University Institute for Advanced Study Synthetic Biology Research Center Shenzhen University Shenzhen People's Republic of China; ^2^ School of Biomedical Engineering Shenzhen University Shenzhen People's Republic of China; ^3^ Shenzhen Institute of Synthetic Biology Shenzhen Institute of Advanced Technology Chinese Academy of Sciences Shenzhen People's Republic of China

**Keywords:** controllable pyroptosis, optogenetics, precise tumor therapy, PyroRACS, synthetic biology

## Abstract

Inducing tumor cell pyroptosis represents a promising anticancer strategy; however, uncontrolled pyroptosis not only restricts the production of viral delivery vectors but also poses a risk of systemic damage, thereby limiting the translational application of pyroptosis‐based therapies. The development of genetic tools that enable precise spatiotemporal control over pyroptosis remains challenging. To address this, we developed PyroRACS, a bioorthogonal optogenetic inducer for precise pyroptosis induction. PyroRACS utilizes an engineered red‐light‐activatable Cre‐ON genetic switch (RACS) to drive the expression of the gasdermin D N‐terminal domain, enabling tunable initiation of pyroptosis without relying on endogenous signaling pathways. We validated robust pyroptosis induction by PyroRACS in multiple cell lines. PyroRACS exhibited high controllability by selectively ablating cancer cells in vitro with precise spatiotemporal resolution. Moreover, its superior controllability enabled the production of the adenovirus vector and allowed “all‐in‐one” delivery of PyroRACS. In a tumor‐bearing mouse model, spatially restricted induction of pyroptosis by PyroRACS resulted in effective tumor suppression, with no detectable systemic toxicity observed under the tested conditions. Collectively, PyroRACS provides a novel optogenetic tool for precise manipulation of pyroptosis, facilitating fundamental research and advancing pyroptosis‐based precision oncology therapeutics.

## Graphical abstract

This study reports a bioorthogonal pyroptosis inducer, PyroRACS. By using a red‐light‐responsive Cre‐On genetic switch to control the expression of highly cytotoxic GSDMD^NT^, PyroRACS enables the artificial induction of pyroptosis with precise spatiotemporal control.

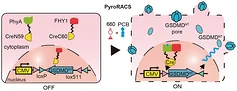

## 1 Introduction

Resistance to cell death endows cancer cells with the capacity to sustain proliferation and evade eradication [1]. As a result, manipulating cell death represents one of the most straightforward and effective strategies for cancer therapy. Distinct cell death modalities have been discovered, among which pyroptosis has demonstrated advantages in tumor treatment due to its inherent immune‐activating properties [2, 3]. Pyroptosis is mediated by conserved gasdermin family proteins including gasdermin A‐E (GSDMA‐GSDME) and pejvakin (PJVK). During pyroptosis, gasdermins undergo caspase cleavage, releasing their N‐terminal domains (GSDM^NT^), which then translocate to the plasma membrane and oligomerize there, culminating in pore formation, cell swelling, and eventual cell lysis [4, 5]. Pyroptotic cells release damage‐associated molecular patterns (DAMPs), such as ATP and high‐mobility group box 1 (HMGB1), which promote immune cell infiltration into tumors, thereby reprogramming the tumor immune microenvironment and enhancing antitumor immunity [6, 7, 8]. Therapeutic strategies for several malignancies, such as lung cancer, breast cancer, and pancreatic cancer, have been shown to benefit from pyroptosis induction [9, 10, 11]. However, uncontrolled pyroptosis not only introduces uncertainty regarding therapeutic efficacy and constrains viral vector production [12], but more importantly, carries the risk of inducing systemic damage [13, 14]. Therefore, achieving controllable induction of pyroptosis represents a critical issue that urgently needs to be addressed in pyroptosis‐based tumor therapy.

Distinct strategies have been developed to achieve controllable induction of pyroptosis, among which nanomedicine‐based platforms represent the predominant approach [15, 16, 17, 18]. For example, an acid‐activatable nano‐photosensitizer was engineered to achieve tumor‐specific and spatiotemporally controlled pyroptosis through acid‐triggered singlet oxygen generation, lipid peroxidation, phospholipase C activation, and subsequent caspase‐3/GSDME signaling [19]. Similarly, an endoplasmic reticulum‐targeting photodynamic conjugate (Indo‐Cy) was shown to induce pyroptosis by precisely damaging the endoplasmic reticulum and activating the NLRP3/caspase‐1/GSDMD signaling cascade [16]. Beyond nanomedicine, bioorthogonal chemistry has recently emerged as a powerful strategy for spatiotemporal regulation of therapeutic processes [20, 21, 22]. Notably, bioorthogonal activation of deep‐red photoredox catalysis enabled mitochondria‐specific activation of caspase‐3/GSDME‐mediated pyroptosis with high spatial precision, demonstrating the feasibility of programmable pyroptosis induction in living systems [23]. In parallel, an optogenetic cell death effector (optoCDE), constructed by fusing the blue‐light‐responsive protein Cry2 to inflammatory caspase‐1, enabled light‐controlled pyroptosis through Cry2‐mediated caspase‐1 oligomerization and subsequent GSDMD cleavage [24].

Despite these advances, current controllable pyroptosis strategies still face several fundamental limitations. First, most approaches ultimately rely on the endogenous expression and activation of key pyroptotic effectors, such as caspase‐1, caspase‐3, GSDMD, or GSDME [15, 19]. However, tumor cells can evade pyroptosis through genetic mutations [8], epigenetic silencing [25], or post‐translational modifications of these signaling components [26]. Moreover, extensive crosstalk among regulated cell death pathways allows the simultaneous activation of apoptosis, necroptosis, or other caspase‐dependent death programs, thereby reducing the specificity and predictability of pyroptosis induction [14, 27]. Furthermore, the reliance of current controllable pyroptosis strategies on sophisticated nanomaterials or bioorthogonal chemical platforms inevitably increases the complexity of material synthesis and chemical engineering [20, 21, 22, 23], thereby hindering their widespread biomedical application.

Direct expression of exogenous pyroptotic executors [12, 28], such as the N‐terminal domain of GSDMD (GSDMD^NT^), offers an alternative strategy to induce pyroptosis independent of endogenous pyroptotic signaling and the crosstalk among regulated cell death pathways. Building on this concept, He et al. developed LiPOP2, a genetically encoded pyroptosis platform that enables light‐controlled activation of an exogenous GSDMD^NT^ executor through the photorelease of caged GSDMD^NT^ upon blue‐light irradiation [29]. Nevertheless, the poor tissue penetration and potential phototoxicity of blue light, together with the intrinsic background cytotoxicity associated with GSDMD^NT^ expression, considerably restrict its versatility and translational potential for in vivo biomedical applications [29, 30].

Herein, we present PyroRACS, the first genetically encoded red‐light‐responsive optogenetic platform that enables programmable pyroptosis through orthogonal control of GSDMD^NT^ expression, independent of endogenous pyroptotic signaling pathways. PyroRACS integrates a red‐light‐activatable genetic switch (RACS) with tightly regulated GSDMD^NT^ expression, allowing precise spatiotemporal control of pyroptosis. Compared with the previously reported blue‐light‐responsive LiPOP2, PyroRACS employs red light as the activating stimulus, providing substantially deeper tissue penetration and reduced phototoxicity, thereby improving its suitability for in vivo applications. Furthermore, the stringent transcriptional regulation conferred by RACS effectively minimizes background GSDMD^NT^ expression, thereby reducing basal cytotoxicity and enabling safer and more reliable control of pyroptosis. Owing to its exogenous execution mechanism and orthogonal gene‐expression control, PyroRACS bypasses endogenous pyroptotic signaling and avoids the uncertainty arising from crosstalk among distinct regulated cell death pathways, thus providing broad applicability across different cellular contexts. We demonstrate that PyroRACS enables robust, spatiotemporally and dose‐controlled induction of pyroptosis with minimal background cytotoxicity in vitro. Moreover, PyroRACS exhibits potent antitumor efficacy in a mouse tumor model. Collectively, this work establishes a general strategy for programmable pyroptosis through red‐light‐responsive optogenetic control, providing a versatile platform for dissecting pyroptosis biology and facilitating the development of pyroptosis‐based precision therapeutics.

## 2 Results

### 2.1 Design of RACS

Initially, we sought to precisely manipulate pyroptosis by expressing GSDMD^NT^ using the red‐light‐inducible transcriptional activation system “REDMAP” [31], capitalizing on both the potent pyroptosis‐inducing capacity of GSDMD^NT^ (Figure S1) and REDMAP's spatiotemporally resolved transcriptional regulation. However, pyroptosis occurred following co‐transfection of REDMAP and GSDMD^NT^ expression plasmids without illumination (Figure S2A,B). Similarly, when employing the Tet‐On system to drive GSDMD^NT^ expression, we observed pyroptosis in the absence of doxycycline (Figure S2C,D). Given that both genetic tools utilize the minimal CMV (miniCMV) core promoter to drive GSDMD^NT^ expression, we hypothesized that basal transcriptional activity of the core promoter triggers GSDMD^NT^‐mediated pyroptosis. Subsequent validation confirmed that GSDMD^NT^ expression driven by core promoters is sufficient to induce pyroptosis (Figure S2E–H). These results indicate that genetic systems reliant on core promoters for GSDMD^NT^ expression face inherent limitations in controllable pyroptosis due to unavoidable basal transcription leakage.

Inspired by the Cre‐dependent FLEX switch system [32], we adopted a loxP‐mediated inversion strategy to suppress basal GSDMD^NT^ expression and thereby mitigate its cytotoxicity. When coupled with an inducible Cre recombinase system, this strategy can enable on‐demand pyroptosis induction. To achieve spatiotemporally precise control, we developed a red‐light‐activated Cre recombinase switch (RACS; Figure 1A). Specifically, RACS was engineered by combining a truncated form of the red‐light photoreceptor phytochrome A (PhyA), its interacting partner, far‐red elongated hypocotyl 1 (FHY1) [31], and a split Cre recombinase. PhyA and FHY1 were selected because they exhibit negligible basal interaction (Figure S3A–C), making them well suited for constructing genetic tools with minimal background activity. In RACS, PhyA is fused to the N‐terminal fragment of Cre (CreN59), whereas FHY1 is fused to the C‐terminal fragment (CreC60). Upon 660‐nm illumination, PhyA bound to the chromophore phycocyanobilin (PCB) is photoactivated and undergoes a conformational change that promotes its interaction with FHY1. The PhyA‐FHY1 complex subsequently translocates into the nucleus via the nuclear localization signal of FHY1, bringing CreN59 and CreC60 into proximity to reconstitute an active Cre recombinase. The reconstituted Cre then catalyzes site‐specific recombination between loxP sites, thereby flipping the loxP‐flanked inverted open reading frame (ORF) of the gene of interest (GOI) into the correct orientation to initiate transcription (Figure 1A).

**FIGURE 1:**
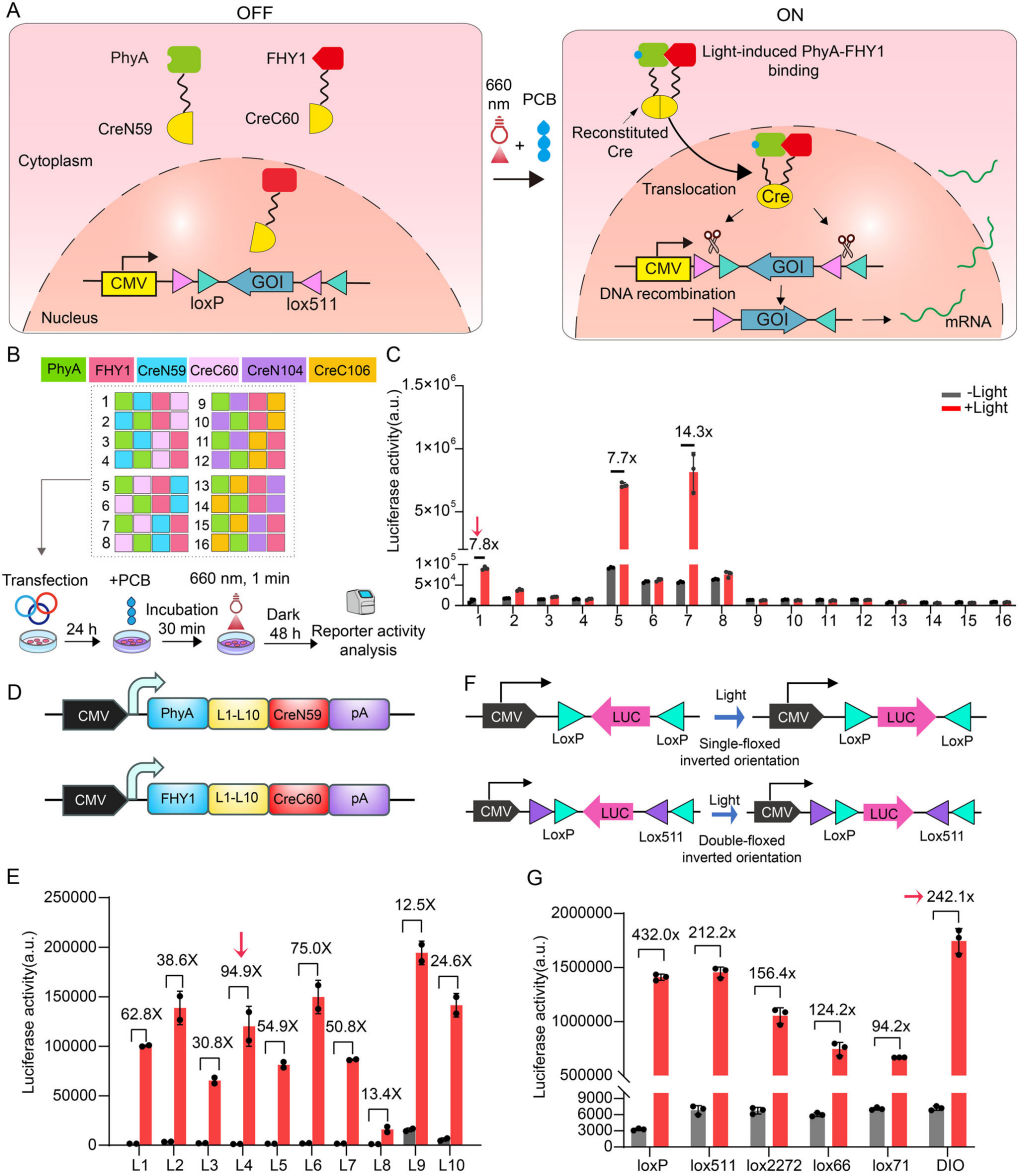
Development of RACS. (A) Schematic of RACS. In the absence of 660‐nm light, the RACS system exists as separate components: cytoplasm‐localized PhyA–CreN59 and FHY1–CreC60, a nucleocytoplasmic shuttling protein. Upon 660‐nm illumination, PCB‐bound PhyA undergoes a conformational transition to its active state, enabling its interaction with FHY1. This light‐induced interaction brings the split Cre fragments into close proximity, leading to reconstitution of an active Cre recombinase. The resulting PhyA–FHY1–Cre complex is then transported into the nucleus with the assistance of FHY1, where Cre catalyzes recombination of the loxP‐flanked inverted open reading frame, thereby placing the gene of interest (GOI) in the correct orientation for transcription and enabling its expression. RACS, red light‐activated Cre‐ON genetic switch. PhyA, phytochrome A. FHY1, Far‐red elongated hypocotyl 1. CreN59, the N‐terminal 59‐amino‐acid fragment of Cre recombinase. CreC60, the C‐terminal fragment of Cre recombinase (residues 60–343). CMV, cytomegalovirus promoter. loxP, Cre recombinase recognition site. lox511, mutant Cre recombinase recognition site. PCB, phycocyanobilin. GOI, gene of interest. (B) Combinatorial screening strategy for RACS components. Sixteen distinct RACS constructs were engineered by varying split‐Cre fragmentation sites (N59/C60 or N104/C106) and fusion protein configurations. Plasmids encoding RACS and a firefly luciferase reporter were transfected into 293T cells. Luciferase activity was quantified 48 h post‐illumination (660 nm, 5 mW/cm^2^, 1 min) to evaluate construct performance. (C) Comparative performance analysis of RACS constructs shown in (B). Data are shown as mean ± SD; *n* = 3 biological replicates. a.u., arbitrary units. (D) Linker design between protein domains. Ten distinct linkers (L1‐L10) with varying flexibility and lengths were inserted between PhyA‐CreN59 or FHY1‐CreC60. pA, polyA signals. (E) Effects of linker properties on RACS activity. 293T cells were transfected with plasmids encoding the PhyA‐linker‐CreN59 and FHY1‐linker‐CreC60 fusions, as well as a luciferase reporter. Luciferase activity was quantified 24 h after light exposure (660 nm, 5 mW/cm^2^, 1 min). Data: mean ± SD; *n* = 3 biological replicates. (F) Design of single‐ and double‐floxed inverted luciferase cassettes. LUC, firefly luciferase. (G) Recombination efficiency of loxP variants. Data: mean ± SD. *n* = 2 biological replicates. DIO, double‐floxed inverted orientation.

To rationally engineer RACS, we systematically optimized the configuration among core components. Specifically, we refined the spatial arrangement between PhyA, FHY1, and split Cre fragments. Prior studies indicate that splitting Cre at residues 59 (N59) and 104 (L104) yields two functional fragment pairs (CreN59/CreC60 and CreN104/CreC106) capable of efficient Cre recombinase reconstitution [33, 34]. Crucially, the domain arrangement of PhyA, FHY1, and split‐Cre fragments significantly influences split‐Cre reconstitution efficiency. Integrating these structural constraints, 16 distinct constructs were generated (Figure 1B). Upon co‐expression with a luciferase reporter, luciferase activity was quantified 48 h post‐illumination. Construct 7 exhibited the highest activity but suffered from elevated background activity. In contrast, construct 1 achieved modest activity while maintaining near‐undetectable background (Figure 1C), prompting its selection (PhyA‐CreN59/FHY1‐CreC60) for iterative refinement.

Linker peptides are essential for modulating synthetic protein functionality by enabling interdomain flexibility and structural rearrangement [35]. To enhance induction efficiency, we screened ten linkers with varying lengths and flexibility connecting PhyA/CreN59 and FHY1/CreC60 (Figure 1D, Table S1). Linker 4 enabled RACS to achieve a 95‐fold induction while preserving minimal background (Figure 1E), and was therefore selected for final assembly. We further evaluated the catalytic efficiency of RACS toward loxP and its derivatives (Figure 1F, Table S2). Luciferase assays confirmed superior recombination activity on both loxP and its variant lox511. Notably, the dual‐floxed inverse orientation (DIO) strategy using loxP/lox511 further enhanced RACS activity while preserving low background activity (Figure 1G). Consequently, DIO was adopted for target gene expression in subsequent studies.

We next investigated the effects of PCB and photostimulation on RACS performance, as both were essential for RACS to activate gene expression [31]. Dose‐response assays demonstrated concentration‐dependent induction, with 5 µM PCB incubation for 30 min achieving maximal activation (Figure S4A–F). Photostimulation tests showed that a single 20‐second pulse of 0.5 mW/cm^2^ 660‐nm light saturated RACS activity (Figure S5A–G), confirming that RACS has photosensitivity comparable to that of the REDMAP system.

### 2.2 RACS for Spatiotemporal Control of Gene Expression

We next profiled RACS‐mediated gene expression regulation, characterizing core performance including induction kinetics, tunability, and spatiotemporal precision. 293T cells were co‐transfected with RACS expression plasmids and a firefly luciferase reporter, with cells harvested at designated time points after illumination for kinetic profiling (Figure 2A). Luciferase activity was detectable as early as 8 h post‐induction, progressively increased, and peaked at 48 h (Figure 2B). Quantitative real‐time PCR revealed luciferase transcript accumulation initiating at 6 h and peaking at 24 h (Figure 2C), aligning with canonical gene expression kinetics. Western blot analysis confirmed luciferase protein expression at 48 h (Figure 2D). RACS exhibited broad cellular compatibility, with tunable induction validated across diverse cell lines (T24 and N2A; Figure S6A,B). Furthermore, mCherry fluorescence imaging provided visual confirmation of RACS activity: signals emerged at 6 h and maximized at 48 h post‐illumination (Figure 2E,F), consistent with luminescence data. No fluorescence was observed in non‐irradiated controls, highlighting the stringency of RACS (Figure 2E).

**FIGURE 2:**
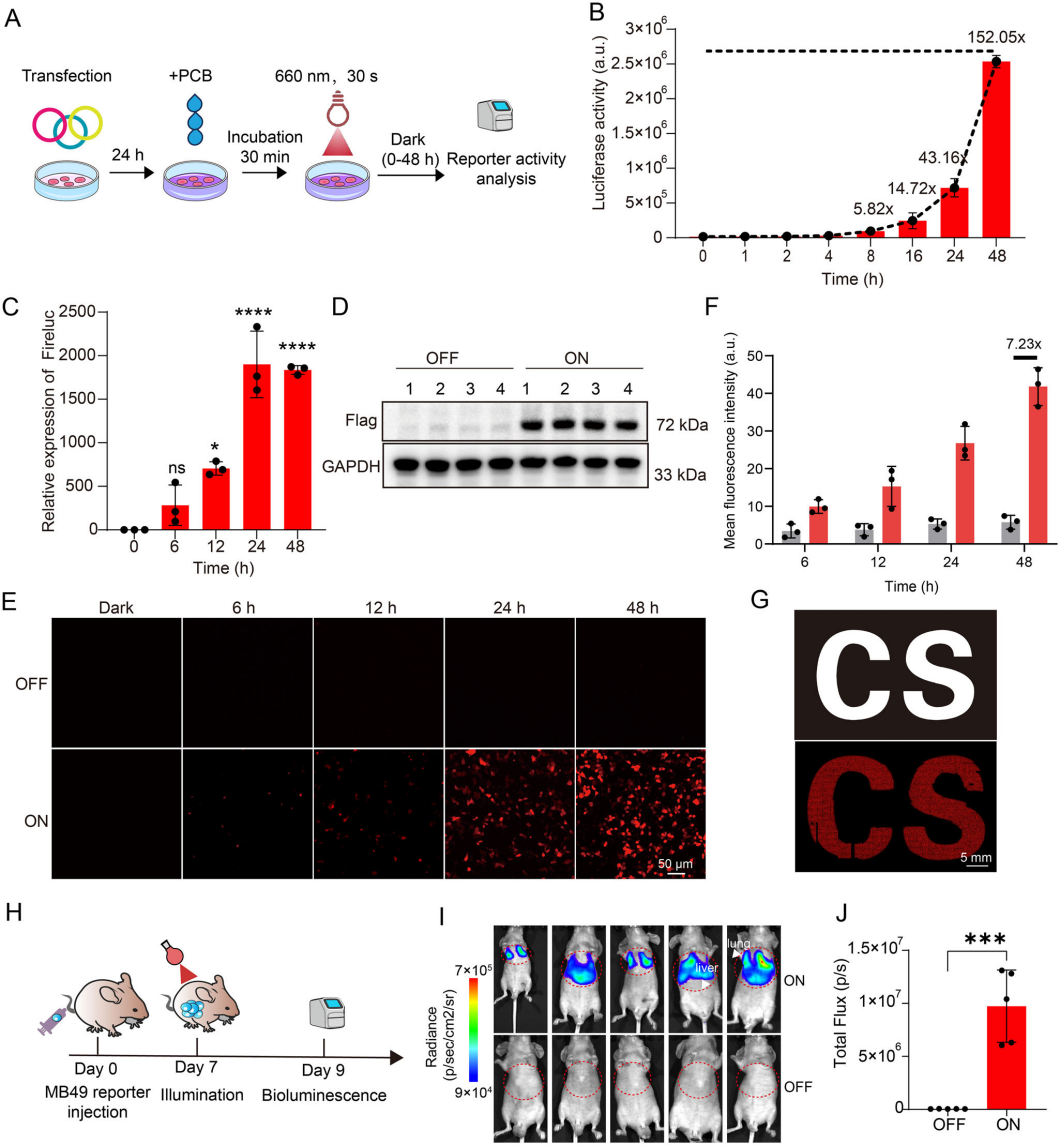
Optogenetic control of gene expression by RACS. (A) Experimental workflow for RACS‐mediated gene expression dynamics. 293T cells were transfected with plasmids encoding RACS and reporters (firefly luciferase and mCherry). At 24 h post‐transfection, cells were treated with 10 µM PCB and exposed to 660‐nm light (1 mW/cm^2^, 30 s) to activate reporter expression. Reporter expression was analyzed at indicated time points within 48 h after illumination. (B) Kinetics of luciferase activity. Data: mean ± SD, *n* = 3 biological replicates. (C) Quantitative RT‐PCR analysis of luciferase mRNA. Data: mean ± SD; one‐way ANOVA with Tukey's multiple comparisons test, ^*^p < 0.05, ^****^p < 0.0001; *n* = 3 biological replicates. (D) Western blot analysis of Flag‐tagged luciferase expression. 293T cells were co‐transfected with plasmids encoding RACS and firefly luciferase. Twenty‐four hours after transfection, cells were incubated with 5 µm PCB and exposed to 660‐nm light (1 mW/cm^2^, 30 s). The illuminated group was designated as ON, whereas the non‐illuminated group (OFF) served as the negative control. Whole‐cell lysates were collected 48 h post‐illumination and probed with anti‐Flag antibody. GAPDH served as a loading control. (E) Confocal microscopy of mCherry expression dynamics. Representative images from three biological replicates; scale bar: 50 µm. (F) Quantification of mCherry mean fluorescence intensity (MFI) in (E). MFI was analyzed using ImageJ. Data: mean ± SD; *n* = 3 biological replicates. (G) Spatially patterned mCherry expression using a photomask. 293T cells were co‐transfected with plasmids encoding RACS and mCherry. At 24 h post‐transfection, 293T cells were illuminated through a “CS”‐patterned photomask (660 nm, 50 µW/cm^2^, 3 min) to induce mCherry expression. Fluorescence images were acquired 48 h post‐illumination. Representative images from two independent experiments. Scale bar: 5 mm. (H) Schematic of in vivo bioluminescence imaging. MB49 cells stably expressing RACS and luciferase were intravenously injected into BALB/c nude mice. At 7 days post‐injection, mice were exposed to 660‐nm light (15 mW/cm^2^, 30 min) or kept in darkness. Bioluminescence imaging was performed 48 h post‐illumination. (I) Representative bioluminescence images of tumor‐bearing mice. Triangles indicate luciferase‐expressing tumor sites. Circles outline the regions of interest (ROIs) used for total flux analysis. (J) Quantification of bioluminescence intensity in (I). Total flux represents the summed bioluminescence intensity within the defined region of interest. Data are presented as mean ± SD; two‐tailed *t*‐test, ^***^p < 0.001; *n* = 5 biological replicates. p/s, photons per second.

A distinctive advantage of optogenetic tools is spatial controllability. To evaluate this, 293T cells were plated in dishes overlaid with a custom photomask (hollow “CS”‐patterned cutouts). After transfection with plasmids expressing RACS and mCherry, cells were irradiated through the dish bottom to region‐specifically activate mCherry expression. Fluorescence imaging revealed mCherry expression precisely constrained to the “CS” pattern (Figure 2G), demonstrating the spatial controllability of RACS.

We further assessed RACS performance in vivo via bioluminescence imaging. A metastatic bladder cancer model was established by tail‐vein injection of MB49 cells stably expressing RACS and luciferase. Bioluminescence imaging was performed 48 h post‐light exposure (Figure 2H). Luminescent signals were significantly enriched in the lungs and liver, thereby confirming the capacity of RACS to regulate gene expression across multiple organs (Figure 2I). Region‐of‐interest (ROI) quantification confirmed RACS‐mediated gene expression in vivo (Figure 2J). Similar results were observed in a subcutaneous tumor‐bearing model (Figure S7A–C). Critically, no background signal was detected in non‐illuminated mice (Figure 2I, Figure S7A), underscoring the stringent regulation of RACS in vivo.

### 2.3 Stringent Optogenetic Regulation of RACS

A defining feature of RACS is its exceptionally low basal leakage. To benchmark stringency, we compared RACS with existing genetic tools. First, we quantified basal activity relative to the minimal promoter, which represents background noise in core promoter‐driven genetic systems. Luciferase assays revealed that RACS exhibited ∼1.6% (1/62) of the basal activity observed with the minimal CMV promoter (Figure 3A). We next compared RACS with the chemically inducible CreERT2 system using EGFP reporters (Figure 3B). Transfected 293T cells were treated with 1 µm 4‐hydroxytamoxifen (4‐OHT), a concentration validated to maximally activate CreERT2 in preliminary studies (Figure S8A,B). Under non‐induced conditions, RACS showed significantly lower background leakage than CreERT2, evidenced by reduced EGFP^+^ cell proportions (0.84% vs. 6.47%; Figure 3D). Transfection‐normalized rates further confirmed this advantage (2.73% vs. 21.11%; Figure S9). Upon induction, despite a 1.85‐fold lower mean fluorescence intensity (MFI), RACS achieved percentages of EGFP^+^ cells comparable to those achieved by CreERT2 (Figure 3E,F, Figure S9B).

**FIGURE 3:**
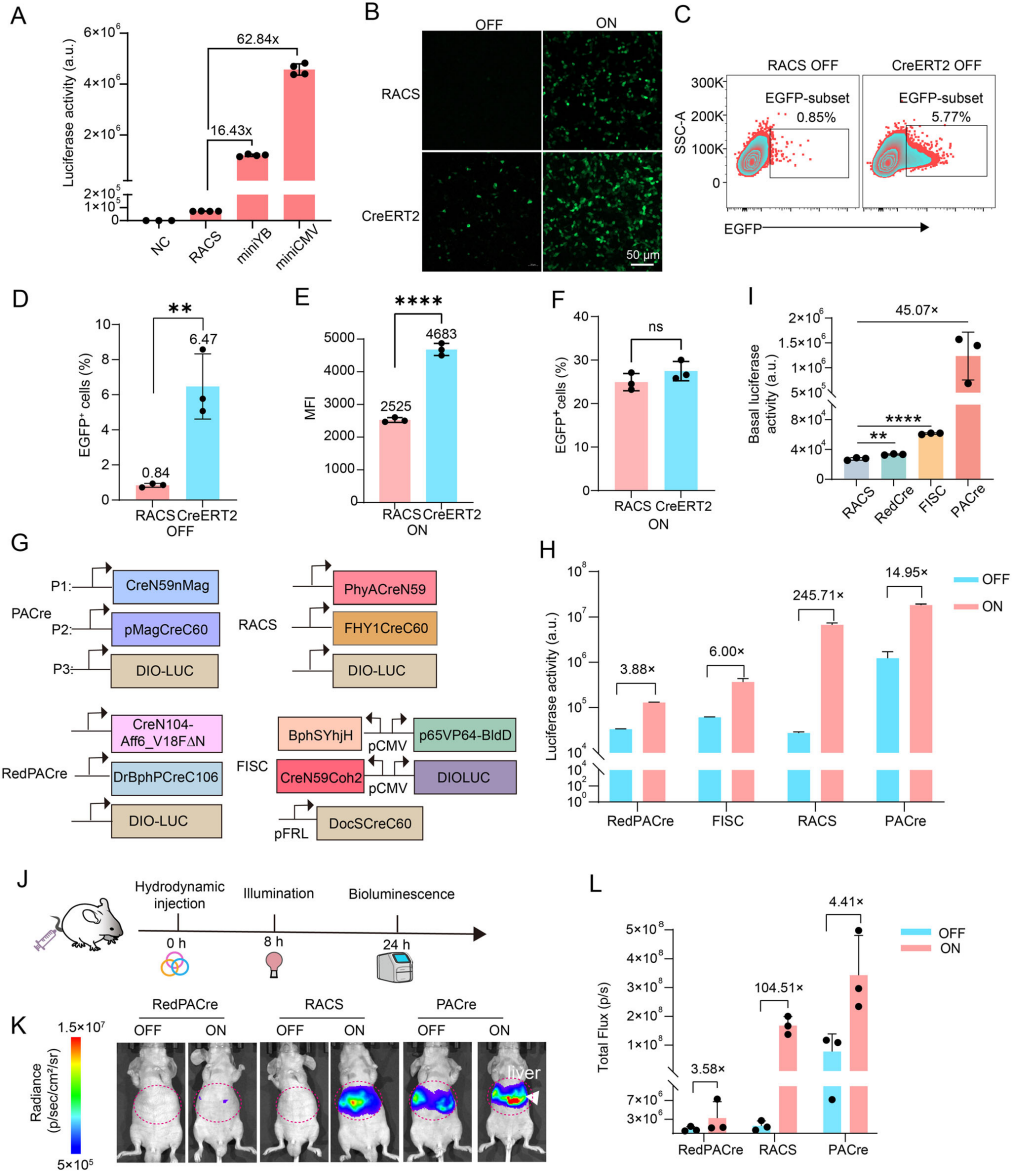
Superior stringency of RACS. (A) Comparison of basal activity between RACS and minimal promoters. 293T cells were co‐transfected with plasmids encoding RACS and a luciferase reporter, or with luciferase reporters driven by minimal promoters. Cells transfected with the empty vector served as the negative control (NC). Luciferase activity was quantified 48 h post‐transfection. miniYB, minimal YB promoter; miniCMV, minimal CMV promoter. (B) Fluorescence imaging of EGFP^+^ 293T cells transfected with RACS or CreERT2 plasmids and an EGFP reporter. Cells were induced with 660‐nm light (1 mW/cm^2^, 30 s) (RACS) or 4‐OH tamoxifen (CreERT2) at 24 h post‐transfection. EGFP expression was quantified by flow cytometry 48 h post‐induction, and non‐induced cells served as controls. Representative confocal images from three independent experiments; scale bar: 50 µm. (C,D) Baseline EGFP leakage in non‐induced cells. Quantification of the proportion of EGFP^+^ cells in the absence of induction by flow cytometry (C,D). (E,F) Post‐induction EGFP expression analysis. Mean fluorescence intensity (MFI) of EGFP (E) and percentage of EGFP^+^ cells (F) were analyzed by flow cytometry. Data represent mean ± SD, *n* = 3 biological replicates; two‐tailed unpaired *t*‐test, ^**^p < 0.01, ^****^p < 0.0001. (G) Plasmid designs for distinct light‐inducible Cre systems (PACre: blue light; RACS/RedPACre: red light; FISC: far‐red light) with luciferase as a reporter. (H) In vitro luciferase induction by optogenetic Cre systems. At 24 h post‐transfection with the plasmids shown in (G), 293T cells were treated under specified illumination conditions (see Table S3) or kept in darkness. Luciferase activity was measured 48 h post‐illumination. Data: mean ± SD, *n* = 3 biological replicates. (I) Comparison of background activity among optogenetic Cre systems. Luciferase activity in 293T cells maintained in the dark was quantified at 48 h post‐transfection. Data represent mean ± SD, *n* = 3 biological replicates; two‐tailed unpaired *t*‐test, ^**^p < 0.01, ^****^p < 0.0001. (J) Experimental design for in vivo performance evaluation. Plasmids encoding optogenetic Cre and luciferase were delivered to mice via hydrodynamic injection. At 8 h post‐injection, mice were illuminated (660 nm, 15 mW/cm^2^, 30 min) and bioluminescence imaging was performed 16 h later. (K) Representative in vivo bioluminescence images. Circles outline the regions of interest used for total flux analysis. (L) Quantification of bioluminescence intensity in (K). Total flux represents the summed bioluminescence intensity within the defined region of interest. Data are presented as mean ± SD, *n* = 3 mice.

To demonstrate competitive advantages, we compared RACS against three photoactivatable Cre systems: blue‐light‐activated PACre [33], red‐light‐responsive RedPACre [34], and far‐red‐inducible FISC (Figure 3G) [36]. Luciferase assays revealed that RACS achieved the highest fold induction (∼246‐fold), attributable to its low basal activity combined with strong inducibility. Although PACre showed maximal activity, its elevated basal expression compromised induction efficiency (Figure 3H,I). Conversely, both RedPACre and FISC exhibited limited induction ranges (Figure 3H). Notably, RACS achieved activation within seconds of illumination, highlighting its rapid response—a distinct advantage over RedPACre and FISC, which require prolonged irradiation (hours) for activation (Table S3). For in vivo validation, we evaluated RACS and comparator systems in mice. Plasmids were delivered via hydrodynamic tail vein injection, followed by light irradiation at 8 h post‐injection (Figure 3J). Bioluminescence imaging at 16 h post‐illumination revealed significant signal enrichment in the livers of RACS and PACre groups, with RACS exhibiting the highest fold change (∼104.5‐fold) (Figure 3K,L). In contrast, RedPACre produced only marginal signals, while FISC showed no detectable bioluminescence (Figure 3L, Figure S10A,B). Consistent with in vitro results, PACre's high background activity severely reduced its induction efficiency (4.4‐fold; Figures 3H,L). Collectively, these results establish RACS as a highly stringent and tunable optogenetic switch, facilitating precise regulation of cytotoxic genes.

### 2.4 PyroRACS‐Encoded Pyroptosis

We next developed a bioorthogonal pyroptosis inducer (“PyroRACS”) by coupling RACS with GSDMD^NT^ expression (Figure 4A). We hypothesized that the bioorthogonal design of PyroRACS enables programmable induction of pyroptosis independent of endogenous gene expression. To validate this hypothesis, we tested PyroRACS in 293T cells that are deficient in inflammatory caspase and gasdermin expression (Figure S11A) [5, 37]. Plasmids encoding RACS and GSDMD^NT^ were co‐transfected, followed by light irradiation to induce GSDMD^NT^ expression. Pyroptosis was evaluated 24 h post‐illumination (Figure 4B). Western blot confirmed GSDMD^NT^ expression following illumination (Figure 4C). In situ Annexin V/propidium iodide (PI) staining revealed that 293T cells underwent pyroptosis, characterized by Annexin V^+^/PI^+^ staining and a transparent, bubble‐like appearance (Figure 4D). Non‐irradiated controls showed no increase in cell death (Figure 4E), confirming the system's superior controllability with minimal background activity. Furthermore, pan‐caspase inhibitor zVAD‐FMK failed to block cell death (Figure 4F), indicating that PyroRACS‐induced pyroptosis is caspase‐independent. Considering that caspases often serve as convergence points for various modes of cell death, the caspase‐independent property of PyroRACS enables pyroptosis‐specific cell death. Indeed, the GSDMD^NT^‐specific inhibitor SCR‐1481B1 significantly attenuated cell death (Figure 4G) [38], demonstrating dependence on GSDMD^NT^ pore formation. Moreover, scanning electron microscopy confirmed the formation of pyroptotic pores in cells following light treatment (Figure 4H).

**FIGURE 4:**
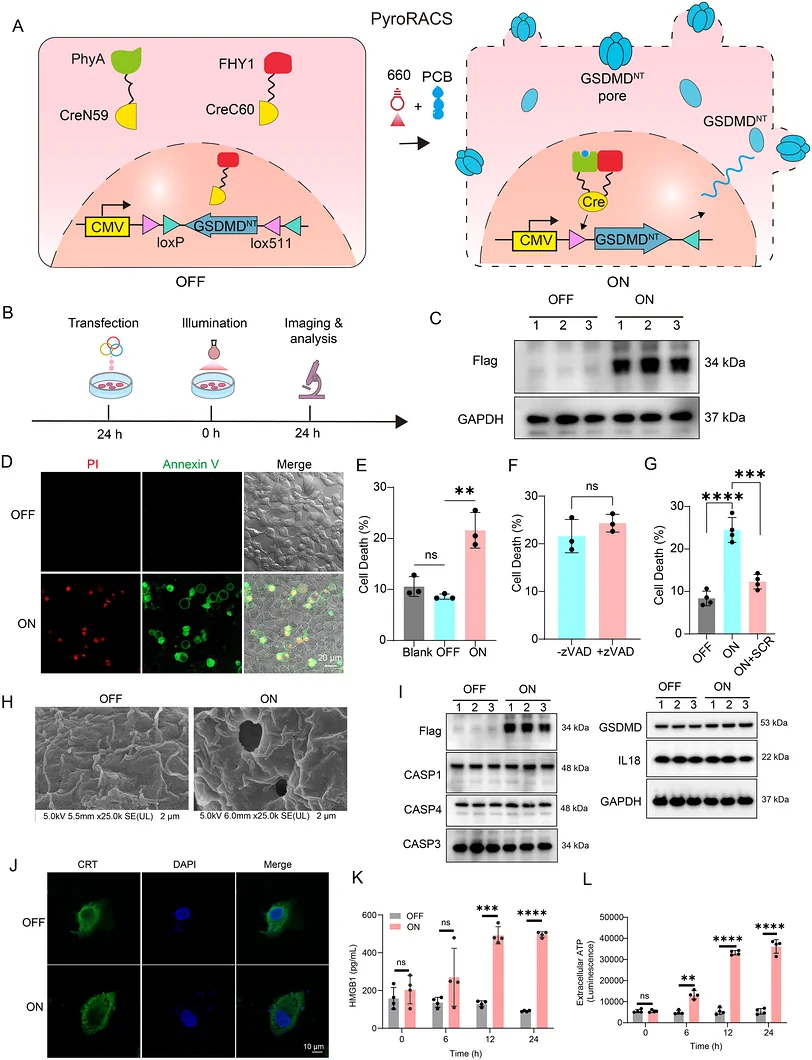
Artificial pyroptosis via PyroRACS. (A) Design of the optogenetic pyroptosis inducer “PyroRACS”. In PyroRACS, 660‐nm illumination activates RACS‐driven GSDMD^NT^ expression, triggering pyroptosis through GSDMD^NT^ oligomerization at the plasma membrane and subsequent pore formation. (B) Experimental design for artificial pyroptosis in 293T cells. 293T cells transfected with plasmids encoding RACS and GSDMD^NT^ were illuminated (660 nm, 1 mW/cm^2^, 30 s) at 24 h post‐transfection or kept in the dark, pyroptosis was analyzed at 24 h post‐illumination. (C) Western blot validation of GSDMD^NT^ expression. 293T cell lysates were prepared at 24 h post‐illumination, and Flag‐tagged GSDMD^NT^ expression was confirmed by immunoblotting using β‐tubulin as a loading control. (D) Fluorescence imaging of pyroptotic 293T cells. Representative of three independent experiments; scale bar: 20 µm. PI, propidium iodide. (E) Quantification of pyroptotic 293T cells by flow cytometry. Data represent mean ± SD, unpaired *t*‐test, *n* = 3 biological replicates. (F,G) Evaluation of pharmacological inhibition of PyroRACS‐mediated pyroptosis. Transfected 293T cells were pretreated for 30 min with 20 µm zVAD‐FMK, a pan‐caspase inhibitor (F), or 20 µM SCR‐1481B1, a GSDMD^NT^ inhibitor (G), prior to illumination. Cell death was quantified by flow cytometry at 24 h post‐illumination. *n* = 3 for (F) and *n* = 4 for (G). (H) Scanning electron microscopy analysis of pyroptotic pore formation. 293T cells were collected for scanning electron microscopy analysis 12 h after light‐induced pyroptosis (660 nm, 1 mW/cm^2^, 30 s), with non‐irradiated cells used as the negative control. (I) Detection of canonical pyroptosis‐related proteins by Western blotting. HeLa cells were transiently transfected with plasmids encoding PyroRACS. At 24 h post‐transfection, cells were irradiated (660 nm, 1 mW/cm^2^, 30 s) to induce pyroptosis. Cell lysates were collected 24 h after irradiation for Western blot analysis. Non‐irradiated cells were used as the negative control. (J–L) Detection of immunogenic cell death markers. HeLa cells were transiently transfected with plasmids encoding PyroRACS. At 24 h post‐transfection, cells were irradiated (660 nm, 1 mW/cm^2^, 30 s) to induce pyroptosis. Cell surface exposure of calreticulin (CRT) was analyzed 12 h after irradiation (J). Culture supernatants were collected at 6, 12, and 24 h after irradiation to assess the time‐dependent release of HMGB1 (K) and ATP (L). Non‐irradiated cells were used as the negative control. Data are presented as mean ± SD; unpaired t‐test, *n* = 4 biological replicates, ^**^ *p* < 0.01, ^***^ *p* < 0.001, ^****^ *p* < 0.0001). HMGB1, high‐mobility group box 1.

Given the intrinsic deficiencies of caspase‐1 and GSDMD expression in 293T cells, we further validated the bioorthogonality of PyroRACS‐induced pyroptosis at the molecular level in HeLa cells. HeLa cells inherently express caspase‐1, GSDMD, IL‐18, and other pyroptosis‐associated markers (Figure S11A). Following 24 h of light stimulation, exogenous Flag‐tagged GSDMD^NT^ expression was detected in HeLa cells. Importantly, no reduction in endogenous full‐length GSDMD protein was observed (Figure 4I), nor was the endogenous GSDMD C‐terminal fragment generated by GSDMD processing detected (Figure S11B), indicating that endogenous GSDMD remained unaffected. Similarly, no processing of pyroptosis‐related inflammatory caspase‐1 or caspase‐4 was observed, and no cleavage of their substrate IL‐18 was detected (Figure 4I, Figure S11B), demonstrating that PyroRACS‐induced pyroptosis does not activate endogenous caspase‐1 or caspase‐4. Additionally, endogenous pro‐caspase‐3 levels remained unchanged, and the necroptotic effector MLKL was not phosphorylated (Figure 4I, Figure S11C), indicating that apoptosis and necroptosis pathways were not triggered.

Pyroptosis is typically accompanied by the release of damage‐associated molecular patterns (DAMPs), including ATP, HMGB1, and lactate dehydrogenase (LDH). Twelve hours after light stimulation, cell‐surface exposure of calreticulin (CRT) was detected by immunofluorescence (Figure 4J), which was further confirmed by flow cytometric analysis showing a significant increase in the proportion of CRT‐positive/7‐aminoactinomycin D (7‐AAD)‐negative cells (Figure S12A,B). In parallel, a time‐dependent release of HMGB1 (Figure 4K), ATP (Figure 4L), and LDH (Figure S12C) into the culture supernatant was detected during pyroptosis. Collectively, these findings indicate that PyroRACS‐induced pyroptosis exhibits the hallmarks of immunogenic cell death. Moreover, IL‐18 levels in the supernatant increased concomitantly with the progression of cell death (Figure S12D); given that no processing of pro‐IL‐18 was detected by Western blot, this release is likely passive, resulting from cell lysis. Collectively, these data demonstrate that PyroRACS enables programmable pyroptosis independent of endogenous gene expression while avoiding crosstalk with other regulated cell death pathways. Notably, RACS‐driven expression of self‐activating caspase‐3 also induced controllable apoptosis (Figure S13A,B), highlighting the versatility of the RACS platform for programmable control of distinct cell death modalities.

### 2.5 Controlled Killing of Tumor Cells by PyroRACS

We next evaluated the ability of PyroRACS to kill tumor cells in a controllable manner. To enable efficient intracellular delivery, we constructed an all‐in‐one adenoviral vector encoding the complete PyroRACS system (Figure 5A). It should be noted that the cytotoxicity of GSDMD^NT^ can lead to the death of viral packaging cells [12], thereby posing a challenge for viral production. The superior controllability of PyroRACS enabled the high‐yield production of its delivery adenovirus without compromising viral titers (Figure 5B).

**FIGURE 5:**
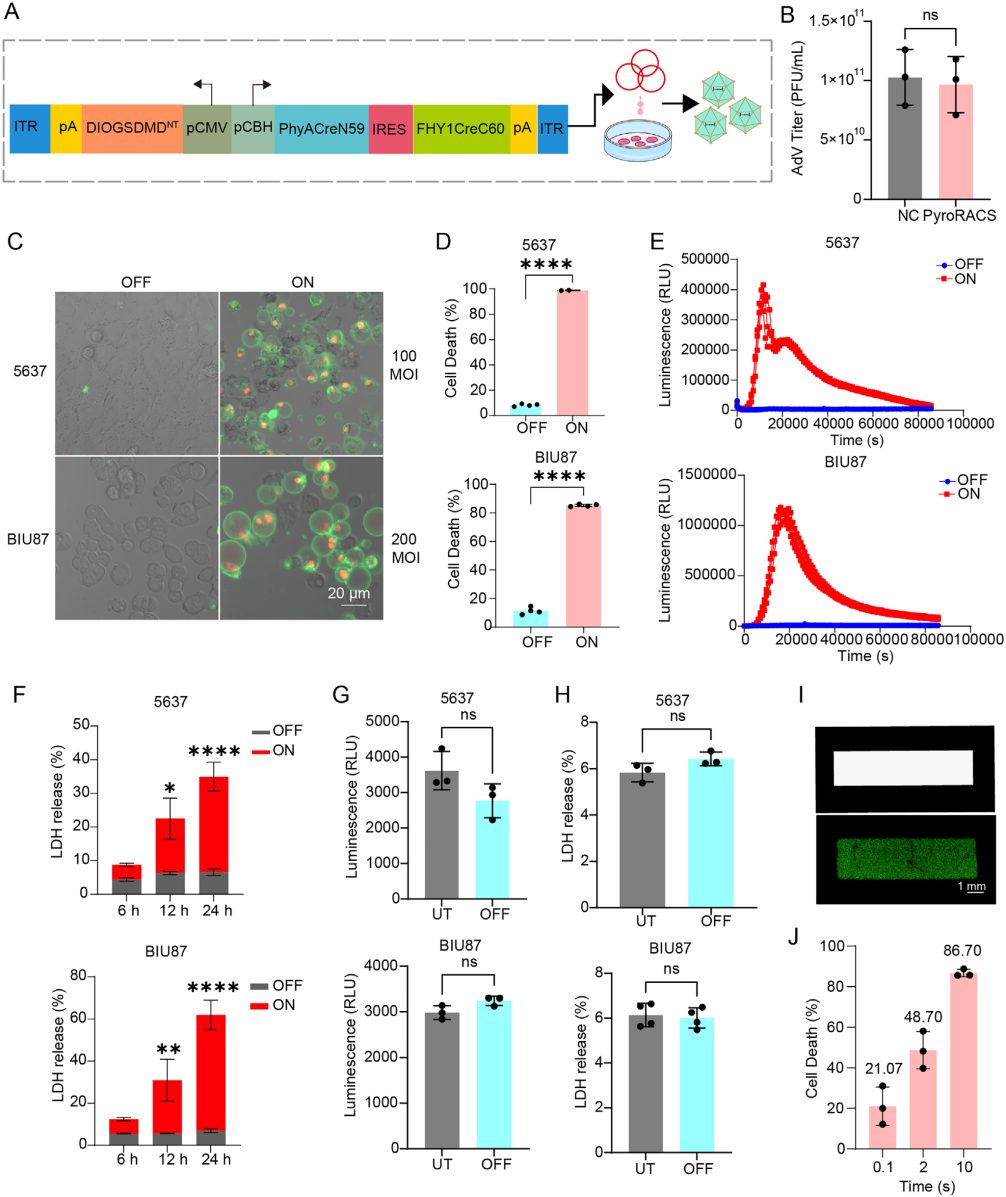
Spatiotemporally controlled tumor cell ablation in vitro. (A) Design of adenoviral vector for all‐in‐one delivery of PyroRACS. Expression cassettes for RACS and GSDMD^NT^ were incorporated into a single adenoviral vector. ITR, inverted terminal repeat; pCMV, cytomegalovirus promoter; pCBH, CBH promoter; IRES, internal ribosome entry site. (B) Titer determination of the adenovirus encoding PyroRACS. Data are presented as mean ± SD; unpaired *t*‐test, *n* = 3 independent replicates. PFU, plaque‐forming units. (C,D) Pyroptosis induction in bladder cancer cells. Bladder cancer cells 5637 and BIU87 were transduced with adenovirus at specified multiplicity of infection (MOI). Pyroptotic cells were stained with Annexin V‐FITC/PI 24 h post‐illumination (660 nm, 1 mW/cm^2^, 30 s). Cell death was quantified by flow cytometry. Representative fluorescence images of pyroptotic 5637 and BIU87 cells (C), scale bar: 20 µm. Flow cytometry quantification of pyroptotic cells (D); data are presented as mean ± SD, unpaired *t*‐test, *n* = 4 biological replicates. (E,F) DAMPs release kinetics during pyroptosis. ATP (E) and LDH (F) levels in culture supernatants were quantified at indicated time points post‐illumination, with non‐illuminated cells serving as negative controls. Data are presented as mean ± SD (F); unpaired *t*‐test, *n* = 4 biological replicates. RLU, relative luminescence units; LDH, lactate dehydrogenase. (G,H) Assessment of cytotoxicity under non‐induced conditions. Following adenoviral transduction, 5637 or BIU87 cells were cultured in the dark for 48 h. The levels of ATP (G) and LDH (H) in the culture supernatant were quantified, with non‐transduced cells (UT) as negative controls. Data: mean ± SD, *n* = 3 (5637) or 4 (BIU87) biological replicates. (I) Spatio‐specific pyroptosis induction. 5637 cells transduced with adenovirus at a MOI of 50. At 48 h post‐transduction, cells were illuminated through a custom‐designed striped photomask to induce localized cell death, followed by SYTOX Green staining 24 h post‐illumination (660 nm, 50 µW/cm^2^, 3 min). The upper panel shows the custom‐designed striped photomask used for patterned illumination. Scale bar: 1 mm; *n* = 2 independent experiments. (J) Light dose‐dependent pyroptosis induction. 5637 cells transduced with adenovirus at a MOI of 100. At 48 h post‐transduction, cells were illuminated (660 nm, 1 mW/cm^2^) for the indicated durations. Cell death was quantified by flow cytometry 24 h after illumination. Data: mean ± SD; unpaired *t*‐test, *n* = 3 biological replicates; ^*^p < 0.05, ^**^ p < 0.01, ^****^ p < 0.0001.

Tumor cells were infected with the PyroRACS adenovirus for 36 h before light irradiation to activate the system. Annexin V/PI staining revealed abundant pyroptotic cells exhibiting a transparent, bubbled morphology with dual‐positive fluorescence post‐irradiation (Figure 5C). Flow cytometric analysis further demonstrated that light activation induced pyroptosis in more than 80% of both 5637 and BIU87 cells (Figure 5D). Concurrently, progressive release of DAMPs, including ATP and LDH, was detected in culture supernatants, correlating with pyroptosis progression (Figure 5E,F).

No pyroptosis or DAMP release was observed in non‐irradiated controls (Figure 5C,G,H), and cell viability remained unaffected after 72 h of culture in the dark (Figure S14A), indicating negligible basal cytotoxicity of PyroRACS in the absence of light activation. Consistent with these findings, no GSDMD^NT^ expression was detected under dark conditions (Figure S14B). Furthermore, evaluation in the mouse neural stem cell line C17.2 (Figure S14C,D) and the human proximal tubular epithelial cell line HK‐2 (Figure S14E,F) demonstrated that PyroRACS had no detectable effects on cell viability or LDH release in the absence of light activation, further supporting its stringent light‐dependent controllability and favorable safety profile.

In addition, spatially specific killing was performed through photomask‐guided irradiation of adenovirus‐transduced 5637 cells. SYTOX Green staining confirmed localized cell death strictly confined to illumination‐patterned zones (Figure 5I). Moreover, dose‐tunable pyroptosis was achieved by modulating irradiation duration (Figure 5J). Altogether, these results demonstrate that PyroRACS enables precise and controllable manipulation of cell death.

### 2.6 PyroRACS for Bladder Cancer Therapy With Enhanced Safety

Due to the high cytotoxicity of GSDM^NT^s, current studies often employ intratumoral delivery of GSDM^NT^s to mitigate the risk of systemic toxicity. However, intratumoral injection itself faces the challenge of drug diffusion (Figure 6A), which may lead to off‐target systemic toxicity. Our results demonstrate that the spatiotemporally controllable nature of optogenetic systems enables tumor‐specific expression of therapeutic payloads (Figure 6A). We next assessed the potential of PyroRACS for the treatment of tumors. To evaluate the antitumor efficacy of PyroRACS, we established syngeneic MB49 bladder carcinoma grafts in immunocompetent C57BL/6 mice. In vitro validation confirmed robust pyroptosis induction in MB49 cells (Figure S15). Tumor‐bearing mice received four cycles of combined intratumoral adenovirus injection and regional tumor illumination (Figure 6B). Analysis of tumor growth kinetics revealed progressive suppression of tumor growth (Figure 6C), with phototherapy cohorts exhibiting 82.5% tumor growth inhibition rate at day 26 post‐inoculation (Figure 6D). Morphological analysis demonstrated a significant reduction in neoplastic mass sizes (Figure 6E), accompanied by an 80.7% decrease in mean tumor weight compared to non‐illuminated controls (Figure 6F). Propidium iodide staining at 24 h post‐illumination confirmed increased cell death in tumors (Figure 6G), validating light‐activated cytotoxicity.

**FIGURE 6:**
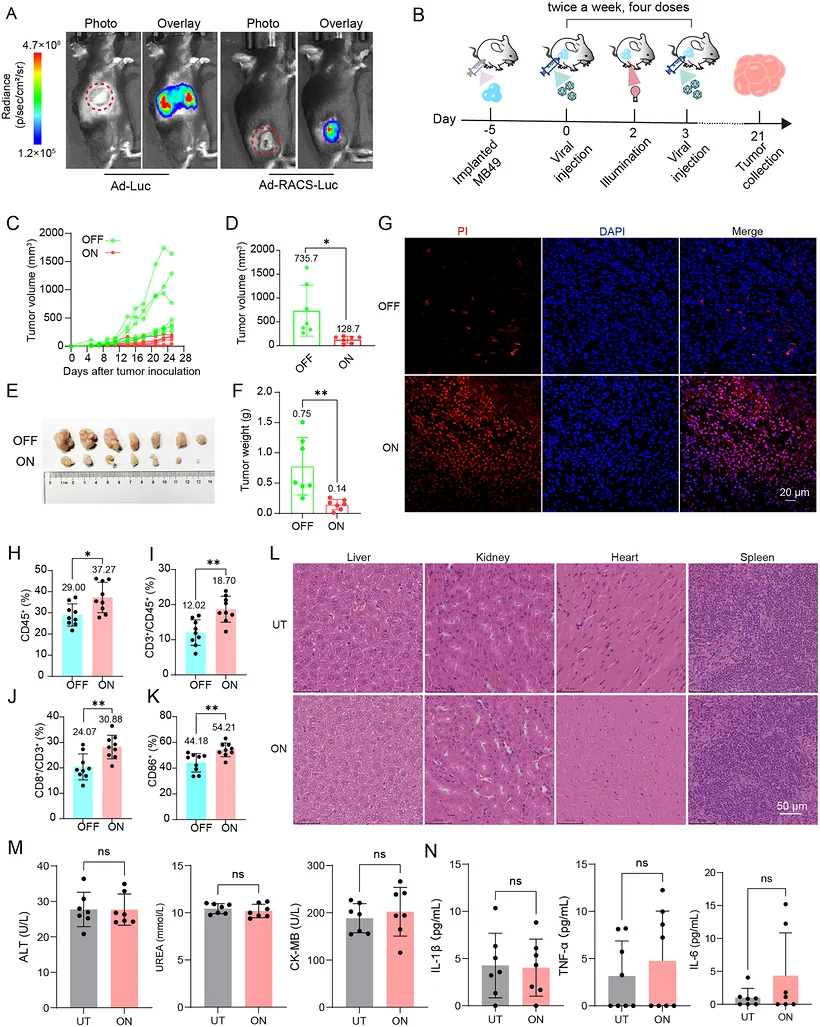
Precision therapy of bladder cancer via PyroRACS. (A) Bioluminescence imaging indicating the spatial distribution of transgene expression. Tumor‐bearing mice received an intratumoral injection of 20 µL adenovirus (2 × 10^9^ PFU). At 36 h post‐injection, regional illumination was performed to induce luciferase expression. Bioluminescence imaging was conducted 24 h post‐illumination (660 nm, 15 mW/cm^2^, 5 min). An adenovirus constitutively expressing luciferase (left panel) was used to indicate diffusion‐mediated extratumoral transgene expression. Representative images are shown from at least three mice per group (left panel, *n* = 3; right panel, *n* = 5). (B) Experimental workflow for in vivo pyroptosis therapy. MB49 cells were subcutaneously engrafted into C57BL/6 mice. At 5 days post‐inoculation, intratumoral injection of adenovirus was performed, followed by spatially confined 660‐nm illumination (15 mW/cm^2^, 5 min) 2 days post‐injection to induce pyroptosis. Therapy was repeated twice weekly (4 cycles). Tumors were harvested at day 21. (C) Tumor growth kinetics. Non‐illuminated (OFF) vs. illuminated (ON) groups, *n* = 7 biological replicates. (D) Endpoint tumor volume (mean ± SD; unpaired *t*‐test, *n* = 7 biological replicates). (E) Excised tumor morphology. (F) Tumor weight quantification. Data: mean ± SD; unpaired *t*‐test, *n* = 7 biological replicates. (G) PI staining of tumor tissue sections. Propidium iodide (PI) was intraperitoneally injected into mice 30 min before euthanasia to label dead cells. Representative images from two independent experiments; scale bar: 20 µm. (H–K) Quantification of immune cell infiltration in tumor. Flow cytometry shows significant increases in: total CD45^+^ leukocytes (H), total CD3^+^ T cells (I), CD8^+^ T lymphocytes (J) and M1‐polarized macrophages (K) in pyroptotic tumors. Data represent mean ± SD; unpaired *t*‐test, *n* = 9 biological replicates; ^*^p < 0.05, ^**^p < 0.01. (L) H&E staining analysis of different organs. Samples were collected after the last round of adenovirus treatment. Untreated (UT) mice were used as a negative control. Representative images from three replicates. Scale bar, 50 µm. (M) Blood chemistry analysis of mice. Following a four‐dose adenovirus treatment, peripheral blood was collected for blood biochemical analysis to assess potential damage to the liver (ALT), kidney (UREA), and heart (CK‐MB). (N) Serum inflammatory cytokine analysis. Peripheral blood was collected after the final adenoviral treatment in mice, and the serum levels of TNF‐α, IL‐6, and IL‐1β were measured by ELISA. Data represent mean ± SD, unpaired *t*‐test, *n* = 7 biological replicates. ALT, alanine aminotransferase; CK‐MB, creatine kinase‐MB.

Given the immunogenic cell death properties of pyroptosis (Figure 4J–L) [39], we assessed immune cell infiltration in tumors after four cycles of therapy. Flow cytometry revealed a 28.52% increase in CD45^+^ leukocytes (Figure 6H), indicating enhanced immune recruitment. Subpopulation analysis demonstrated significant elevation of CD3^+^ T cells (+55.57%, Figure 6I) and CD8^+^ T lymphocytes (+28.29%, Figure 6J), confirming pyroptosis‐driven T lymphocyte infiltration. Furthermore, M1‐polarized macrophages (F4/80^+^CD86^+^) increased by 22.70% (Figure 6K), validating amplification of pro‐inflammatory myeloid responses. These results demonstrate that PyroRACS kills tumor cells while simultaneously converting immunologically cold tumors into hot tumors, thereby enhancing the efficacy of cancer therapy.

Spatiotemporally restricted induction of pyroptosis is expected to improve therapeutic safety by minimizing off‐target cytotoxicity. We next systematically evaluated the biosafety profile of PyroRACS‐mediated tumor therapy. Dynamic monitoring of body weight during treatment revealed no significant changes in the mice (Figure S16A). Histopathological examination of major organs, including the heart, liver, spleen, and kidneys, showed no detectable tissue damage (Figure 6L). Consistently, serum biochemical assays detected no abnormal elevations in ALT, AST, UREA, CREA, or CK‐MB (Figure 6 m, Figure S16B). Moreover, peripheral blood levels of TNF‐α, IL‐6, and IL‐1β remained unchanged (Figure 6N), indicating the absence of excessive systemic inflammatory responses. Collectively, these findings indicate that locally delivered PyroRACS exhibits a favorable biosafety profile in the present experimental setting while maintaining potent antitumor efficacy.

## 3 Discussion

Pyroptosis‐based cancer therapy has been fundamentally constrained by the inability to precisely control pyroptosis induction. Such uncontrollability not only hampers the production of viral vectors carrying pyroptosis‐inducing cargos but also compromises therapeutic precision and increases the risk of off‐target toxicity, underscoring the urgent need for strategies that enable precise spatiotemporal regulation of pyroptosis. To address this challenge, we developed PyroRACS, a genetically encodable, red light‐controlled, bioorthogonal pyroptosis induction system that enables programmable pyroptosis with precise spatiotemporal control in cells with diverse biological contexts. Leveraging this high degree of controllability, we successfully produced an all‐in‐one adenoviral vector encoding the complete PyroRACS genetic circuit. Importantly, intratumoral delivery of PyroRACS followed by localized red‐light irradiation achieved robust tumor suppression while maintaining a favorable safety profile, highlighting the therapeutic potential of PyroRACS for cancer treatment.

Controllable pyroptosis entails two critical aspects: cell death should proceed via pyroptosis rather than alternative death pathways, and its initiation should be precisely controlled. Current strategies largely induce pyroptosis by activating endogenous pyroptotic signaling pathways in tumor cells. For instance, chemotherapeutic agents such as cisplatin and paclitaxel can induce pyroptosis in GSDME‐positive tumors [40], whereas nanosensitizer‐based photodynamic therapy selectively triggers pyroptosis in malignancies expressing GSDME [19]. However, pyroptosis resistance may arise from deficient gasdermin expression, such as GSDME promoter hypermethylation [25]. Furthermore, extensive crosstalk among regulated cell death pathways introduces uncertainty in pyroptotic outcomes, potentially resulting in mixed cell death modalities rather than bona fide pyroptosis [14, 27]. In contrast, PyroRACS induces pyroptosis through the exogenous expression of GSDMD^NT^, thereby bypassing reliance on endogenous pyroptosis machinery. Consistent with this design, PyroRACS effectively induced pyroptosis across diverse cell types with distinct biological backgrounds, including 293T cells deficient in gasdermin expression (Figures 4, 5, Figure S14). Moreover, because GSDMD^NT^ is expressed directly without requiring upstream caspase activation, PyroRACS largely avoids crosstalk with other regulated cell death pathways, ensuring highly specific and orthogonal induction of pyroptosis.

Given the potent cytotoxicity of GSDM^NT^s, achieving precise control over GSDM^NT^ expression and GSDM^NT^‐mediated pyroptosis remains a major challenge. To tackle this, Wang et al. developed a bioorthogonal pyroptosis induction strategy in which the tumor‐imaging probe Phe‐BF catalyzes desilylation to release GSDMA3^NT^ from nanoparticle‐GSDMA3 nanocomplexes, thereby inducing tumor cell pyroptosis [3]. However, the preparation of NP‐GSDMA3 involves complex procedures including purification and chemical modification of GSDMA3 (NT+CT) proteins, limiting the general applicability of this approach. Moreover, the nanoparticle‐based strategy suffers from insufficient tumor targeting and restricted spatiotemporal controllability, which increases the risk of off‐target cytotoxicity. In contrast, PyroRACS provides a genetically encoded, bioorthogonal pyroptosis platform that couples optogenetic regulation to GSDMD^NT^ expression, thereby enabling programmable pyroptosis with high spatiotemporal precision. A conceptually related strategy, LiPOP2, employs the LOV2 photosensory module to directly uncage the caged GSDMD^NT^ protein upon blue‐light illumination, enabling rapid pyroptosis induction [29]. Nevertheless, the limited tissue penetration and phototoxicity of blue light, together with basal cytotoxicity caused by the background activity of LiPOP2, restrict its broader application. By comparison, PyroRACS employs red light to transcriptionally control GSDMD^NT^ expression, providing deeper tissue penetration, improved biocompatibility, and stringent regulation of pyroptosis induction. Consistent with this design, negligible basal cytotoxicity was observed in the absence of illumination (Figure 5G,H, Figure S14), supporting the utility of PyroRACS as a highly controllable pyroptosis inducer.

Beyond precise regulation of pyroptosis induction, efficient delivery of GSDM^NT^s presents another major challenge. Because GSDM^NT^s are intrinsically cytotoxic, their expression in viral packaging cells, such as 293T cells, prevents efficient viral vector production. To overcome this limitation, Lu et al. developed a dual‐AAV strategy in which one vector encodes Cre recombinase and the other carries an inverted GSDM^NT^ expression cassette, thereby enabling viral production [12]. Although intratumoral administration reduces systemic exposure, local dissemination of viral vectors may still lead to off‐target transgene expression in surrounding normal tissues. In contrast, PyroRACS combines localized vector delivery with spatially confined red‐light activation to provide two layers of spatial regulation. We demonstrated that transgene expression was strictly restricted to the illuminated tumor region following intratumoral administration (Figure 6A). This dual spatial‐gating strategy effectively minimized local off‐target effects while preserving robust antitumor efficacy, thereby substantially improving the safety profile of pyroptosis‐based cancer therapy.

Compared with the blue light‐responsive pyroptosis system LiPOP2, which directly activates caged GSDMD^NT^ to induce rapid pyroptosis upon illumination, PyroRACS operates through a sequential process involving DNA recombination, transcription, and translation before GSDMD^NT^ is produced. As a result, PyroRACS displays slower activation kinetics and may be less suitable for applications requiring immediate pyroptosis induction. In addition, because PyroRACS is based on a DNA recombination–transcription‐coupled genetic circuit, its implementation depends on DNA‐based gene delivery systems. Consequently, the current design is not directly compatible with transient mRNA delivery strategies, which have recently attracted considerable attention owing to their favorable safety profiles and translational potential. To enable one‐vector delivery of the complete genetic circuit, we employed an adenoviral vector in this study. However, owing to the inherent immunogenicity of adenoviral vectors [41], we did not assess the feasibility of systemic delivery of PyroRACS combined with localized illumination for safe tumor therapy. Moreover, although PyroRACS exhibited negligible basal cytotoxicity in non‐tumor‐derived cells under the experimental conditions tested (Figure S14C–F), its biosafety has yet to be evaluated in normal tissues and at the whole‐organism level. Future studies using delivery platforms with improved biosafety, such as AAV, may facilitate comprehensive multi‐organ safety evaluation while further expanding the therapeutic applicability of PyroRACS.

More broadly, achieving systemic yet tumor‐selective delivery remains one of the principal challenges facing GSDM^NT^‐based therapeutics, particularly for deep‐seated malignancies. This limitation arises from the potent, non‐selective cytotoxicity of GSDM^NT^ together with the broad tissue tropism of currently available delivery vehicles, including AAV [42]. We anticipate that the integration of PyroRACS with safer systemic delivery platforms, such as engineered AAVs or tumor‐targeted nanoparticle vectors, together with regional light irradiation, could provide a promising strategy for the precise treatment of non‐superficial tumors while minimizing off‐target toxicity. Such advances may further broaden the translational potential of optogenetically controlled pyroptosis for cancer therapy.

## 4 Conclusion

In this study, we developed PyroRACS, a red‐light‐controlled optogenetic pyroptosis inducer that integrates the stringent and tunable RACS switch with the cytotoxic effector GSDMD^NT^. PyroRACS enables precise spatiotemporal induction of pyroptosis independent of endogenous signaling pathways, effectively triggering pyroptosis even in gasdermin‐deficient cells while avoiding crosstalk with alternative death pathways. Its superior controllability and minimal basal leakage not only overcome challenges in viral vector production but also reduce the risk of off‐target tissue damage during in vivo delivery, thereby potentially improving the safety profile. Functionally, PyroRACS demonstrated robust in vitro pyroptosis induction, localized and tunable tumor cell ablation, and potent antitumor efficacy in vivo with enhanced immune cell infiltration, without evidence of detectable systemic toxicity in the treated mice. Collectively, PyroRACS establishes a versatile platform for controllable pyroptosis, providing a powerful tool for mechanistic studies and advancing the development of precision oncology therapies.

## 5 Experimental Section

### 5.1 Plasmids

Plasmids used in this study are detailed in Table S4. Coding sequences for GSDMA3^NT^, GSDMB^NT^, GSDMD^NT^, GSDME^NT^, mCherry, and luciferase were amplified by high‐fidelity PCR from plasmids provided by Youbio Biological Technology Co., Ltd. (Changsha, China). PCR products were subsequently cloned into the expression vectors using the Hieff Clone One Step Cloning Kit (YEASEN, Cat. 10912ES10). All other recombinant constructs were synthesized commercially by Genscript Biotech Corp. (Nanjing, China). All plasmid constructs were verified by Sanger sequencing. Key DNA sequences are provided in Supplementary Data.

### 5.2 Cell Culture and Transfection

Human embryonic kidney 293T (HEK293T) cells, human bladder cancer cells (5637 and T24), and mouse neuroblastoma Neuro‐2a (N2A) cells were obtained from the American Type Culture Collection (ATCC). 293A cells were purchased from Invitrogen (Cat. R70507). Mouse bladder cancer cells (MB49) were acquired from EK‐Bioscience Biotechnology Co., Ltd. (Shanghai, China). Except for 5637 cells, which were cultured in RPMI 1640 medium (Gibco, Cat. C11875500BT), all other cell lines were maintained in DMEM medium (Gibco, Cat. C11995500BT) supplemented with 10% fetal bovine serum (Gibco, Cat. 10437028) and 1% penicillin‐streptomycin (Gibco, Cat. 15140122). Cells were incubated at 37°C under 5% CO_2_ atmosphere.

All cell lines were transfected with Lipofectamine 3000 (Invitrogen). Briefly, 5 × 10^5^ cells were plated per well in a 12‐well cell culture plate and cultured for 18 h before transfection. 1200 ng plasmids were used per transfection. For co‐transfection experiments, different plasmids were transfected in an equal ratio. The transfection complexes were prepared according to the manufacturer's protocol, comprising Lipofectamine 3000 Reagent, P3000 Reagent, and target plasmids. The mixture was then added dropwise to the cells and immediately returned to the incubator.

### 5.3 Adenovirus Production and Infection

To achieve efficient delivery, the coding sequences of RACS and GSDMD^NT^ were integrated into a single adenoviral expression vector (pAd/PL‐DEST). Adenovirus production was performed according to the manufacturer's instructions for the Adenoviral Promoterless Gateway Expression Kit (Invitrogen, Cat. K494000). Briefly, the RACS and GSDMD^NT^ coding sequences, along with their expression regulatory elements, were cloned into the promoterless pAd‐DEST vector via Gateway cloning to generate the adenoviral expression construct. Twenty‐four hours prior to virus production, 2 × 10^6^ 293A cells were seeded into 6‐cm culture dishes to ensure 70% confluency at the time of transfection. The medium was replaced with fresh complete medium 24 h post‐transfection, with appropriate medium supplementation during the incubation period. Cells exhibiting late‐stage cytopathic effect were harvested 10–14 days post‐transfection to obtain the primary adenovirus. For virus amplification, the primary virus was used to sequentially infect T75–T175 cultures. The final crude viral supernatant was concentrated and purified using the Adeno‐X Maxi Purification Kit (Clontech, Cat. 631533), yielding adenovirus with a titer of 1 × 10^11^ PFU/mL for subsequent in vitro infections or in vivo therapies.

For in vitro adenoviral infection, cells were infected with recombinant adenovirus at the specified multiplicity of infection (MOI) using a reduced‐volume adsorption protocol (virus diluted in 50% of standard culture volume). After 6 h of adsorption, the medium was replaced with fresh complete medium. An additional full medium change was performed at 24 h post‐infection to maintain nutrient supply and remove residual virus particles. For intratumoral adenoviral delivery, adenovirus suspensions (10 µL containing 1 × 10^9^ PFU) were injected into tumors via a microsyringe using a multi‐point injection strategy. Injections were administered twice weekly at 72‐hour intervals for a total of four treatments.

### 5.4 Light‐Induced Gene Expression

Light of different wavelengths was generated using LED light sources (Shenzhen Firefly Technology Co., Ltd) with a wavelength accuracy of ± 20 nm. Different light intensities were achieved by adjusting the input voltage and calibrated using a spectrometer (OHSP‐350C, Zhejiang Hopoo Light & Color Technology Co., Ltd; Figure S17A). Unless otherwise specified, in vitro systems (cells treated with 5 µM PCB) and in vivo models (mice administered 10 mg/kg PCB via intraperitoneal injection) were pre‐incubated with PCB for 30 min prior to illumination. Subsequent photostimulation was performed under parameter‐specific illumination conditions as detailed in Table S3. Specifically, mice were housed in custom‐designed transparent acrylic chambers (length × width × height: 140 × 110 × 210 mm) to facilitate unrestricted movement (Figure S17B). During prolonged irradiation protocols, mice were permitted 30‐minute intervals for ad libitum feeding/drinking every 6 h to minimize physiological stress.

### 5.5 Spatial Control of RACS‐Mediated Transgene Expression

293T or 5637 cells were plated in 6‐cm culture dishes at 50% confluency. A hollow photomask with customized letter patterns was placed at the bottom of the dish to enable region‐selective light transmission. After 24 h of transient transfection (293T) or 48 h of adenoviral infection (5637), cells were irradiated through the dish bottom (660 nm, 50 µW/cm^2^, 3 min) to initiate gene expression. 293T cells were subjected to fluorescence imaging at 48 h post‐irradiation using an LSM 800 confocal microscope. For spatial profiling of pyroptosis induction, SYTOX Green (Invitrogen, Cat. R37109) was added dropwise to the culture medium of 5637 cells at 24 h post‐irradiation, following the manufacturer's instructions. After 10 min of incubation for dead cell staining, fluorescence imaging was immediately performed.

### 5.6 Luciferase Reporter Assay

Luciferase activity was measured using the Luciferase Reporter Gene Assay Kit (YEASEN, 11401ES80) following the manufacturer's protocol. Specifically, at the experimental endpoint, the culture supernatant of pretreated 293T cells was removed. Then, 300 µL lysis buffer per well (for 12‐well plates) was added, followed by incubation on ice for 5 min to ensure complete cell lysis. The lysate was centrifuged at 12,000 g for 1 min at 4°C to pellet cellular debris, and the supernatant was collected. Subsequently, 20 µL of the supernatant was transferred to a black opaque 96‐well luminescence plate. Next, 100 µL firefly luciferase detection reagent was added to each well. After vortexing for 15 s, chemiluminescence signal was immediately quantified using a multimode plate reader (PerkinElmer, VICTOR Nivo).

### 5.7 Cell Viability Assay

5637 cells (5 × 10^4^) were seeded in 96‐well plates 12 h prior to infection. Cells were then infected with PyroRACS‐expressing adenovirus at a multiplicity of infection (MOI) of 50. Twelve hours post‐infection, the medium was replaced with fresh complete medium or complete medium supplemented with 5 µm phycocyanobilin (PCB), which was defined as time zero. Cell viability was subsequently measured every 12 or 24 h. For the assay, plates were equilibrated to room temperature for 30 min before adding an equal volume of CellTiter‐Glo 2.0 reagent (Promega, Cat. G7570). Plates were shaken for 2 min to lyse the cells, followed by a 10‐minute incubation at room temperature. Luminescence was then recorded to quantify cell viability.

### 5.8 Western Blotting

Protein expression was analyzed by Western blotting following established protocols. Briefly, approximately 1 × 10^6^ 293T cells were collected, lysed using RIPA buffer (Thermo Scientific, Cat. 89901), and centrifuged at 4°C to obtain cell extracts. Loading buffer was added to the extracts, followed by boiling for protein denaturation. Total protein (20 µg) was separated via 12% polyacrylamide gel electrophoresis (SDS‐PAGE) and transferred onto a 0.22‐µm PVDF membrane (Millipore). The membrane was blocked with 5% bovine serum albumin for 1 h at room temperature (RT), then incubated with diluted primary antibody overnight at 4°C. Unbound primary antibody was removed by washing three times with 0.1% TBST buffer. The membrane was subsequently incubated with a species‐specific HRP‐conjugated secondary antibody for 1 h at RT. After three additional TBST washes, protein bands were visualized using enhanced chemiluminescence reagent (Epizyme, Cat. SQ202L) and imaged with an Amersham Imager 680 (Cytiva) with exposure times optimized to avoid saturation. The following antibodies were used: Anti‐FLAG M2 mouse monoclonal antibody (Sigma–Aldrich, Cat. F1804; 1:1000), Anti‐GSDMD rabbit monoclonal antibody (Abcam, Cat. ab324385; 1:1000), Anti‐GAPDH mouse monoclonal antibody (TransGen Biotech, Cat. HC301; 1:3000), Anti‐Caspase‐1 rabbit monoclonal antibody (Proteintech, Cat. 81482‐1‐RR; 1:3000), Anti‐Caspase‐3 rabbit monoclonal antibody (CST, Cat. 14220; 1:1000), Anti‐Caspase‐4 rabbit monoclonal antibody (CST, Cat. 42264; 1:1000), Anti‐MLKL mouse monoclonal antibody (CST, Cat. 26539; 1:1000), Anti‐phospho‐MLKL (Ser358) rabbit monoclonal antibody (CST, Cat. 91689; 1:1000), Anti‐IL‐1β rabbit monoclonal antibody (Selleck, Cat. F3061; 1:1000), Anti‐IL‐18 rabbit monoclonal antibody (Selleck, Cat. F2146; 1:1000), Goat anti‐mouse IgG, HRP‐conjugated (TransGen Biotech, Cat. HS201; 1:3000), Goat anti‐rabbit IgG, HRP‐conjugated (TransGen Biotech, Cat. HS101; 1:3000).

### 5.9 Immunofluorescence Staining

HeLa cells (1 × 10^5^) were seeded in 6‐well glass‐bottom confocal dishes and cultured for 12 h before transfection with 2 µg of PyroRACS‐expressing plasmids using Lipofectamine 3000. Twenty‐four hours post‐transfection, the medium was replaced, and cells were subjected to light stimulation to induce pyroptosis. Twelve hours after illumination, the culture medium was carefully removed, and cells were washed with PBS, followed by fixation with 4% paraformaldehyde at room temperature for 20 min. After three PBS washes, cells were permeabilized with 0.25% PBST for 20 min at room temperature. Following another three PBS washes, cells were blocked with 10% goat serum for 1 h at room temperature. Cells were then incubated overnight at 4°C with the primary antibody against calreticulin (Selleck, Cat. F0472; 1:200). After three additional PBS washes, cells were incubated with Alexa Fluor 488‐conjugated goat anti‐rabbit secondary antibody (ThermoFisher, Cat. A‐11008; 1:1000) for 1 h at room temperature. Following three final PBS washes, mounting medium containing DAPI was added, and fluorescence imaging was subsequently performed.

### 5.10 Flow Cytometric Analysis of Cell‐Surface Calreticulin Exposure

HeLa cells (1 × 10^5^ cells per well) were seeded in 12‐well plates and cultured for 12 h before transfection with 1 µg of PyroRACS‐expressing plasmid using Lipofectamine 3000. Twenty‐four hours after transfection, the culture medium was replaced with complete medium supplemented with 5 µM PCB, followed by 660‐nm light irradiation to induce pyroptosis. Twelve hours after light stimulation, both floating and adherent cells were collected. Floating cells in the culture supernatant were harvested directly, whereas adherent cells were detached by incubation with PBS containing 2 mM EDTA at 37°C for 3–5 min. The collected cells were pelleted by centrifugation at 500 × g for 5 min at 4°C, washed once with flow cytometry staining buffer, and stained without fixation on ice with PE‐conjugated anti‐calreticulin antibody (Cell Signaling Technology, Cat. 19780) for 20 min. Subsequently, 7‐AAD was added, and cells were incubated for an additional 10 min. After two washes with staining buffer, cells were resuspended in 500 µL of staining buffer and analyzed by flow cytometry. Cells exhibiting cell‐surface calreticulin exposure were identified as the CRT‐positive/7‐AAD‐negative population.

### 5.11 Quantitative Real‐Time PCR

Total RNA was isolated from cells using the MolPure Flash Cell/Tissue Total RNA Kit (YEASEN, Cat. 19221ES50), following the manufacturer's protocol. Briefly, 1 × 10^6^ cells were lysed, and RNA was purified within 15 min without phenol/chloroform extraction. The extracted RNA (1 µg) was then reverse‐transcribed into cDNA using Hifair III first Strand cDNA Synthesis SuperMix (YEASEN, Cat. 11141ES60) in a 20 µL reaction system. The reaction conditions were: 42°C for 2 min (genomic DNA removal), 25°C for 5 min (primer annealing), 55°C for 15 min (reverse transcription), and 85°C for 5 min (enzyme inactivation). qPCR was performed with Hieff qPCR SYBR Green Master Mix (YEASEN, Cat. 11202ES08) on a QuantStudio 3 Real‐Time PCR System (Thermo Fisher Scientific). GAPDH was used as a loading control. Primer sequences are listed as below: Luciferase‐F: AACATTTCGCAGCCTACCGTA, Luciferase‐R: TGACGAACGTGTACATCGACT; human GAPDH‐F: GATTCCACCCATGGCAAATTC, human GAPDH‐R: CTGGAAGATGGTGATGGGATT.

### 5.12 Annexin V/PI Staining

For in situ dead cell staining, 25 µL of Annexin V‐FITC (YEASEN, Cat. 40302ES60) or Annexin V‐YSFluor 647 (YEASEN, Cat. 40304ES60) and 25 µL of PI were added to each well of a 12‐well plate. After incubation at RT in the dark for 10 min, confocal microscopy imaging was performed immediately (ZEISS, LSM 800).

For flow cytometric quantification of dead cells, culture supernatants and trypsin‐digested cells were centrifuged and collected into 1.5 mL Eppendorf tubes. The collected cells were resuspended in 100 µL of binding buffer, and 5 µL of Annexin V‐FITC and 5 µL of PI were added to each tube of cell suspension. After incubation at RT in the dark for 10 min, 400 µL of pre‐chilled binding buffer was added. After resuspension and mixing, samples were analyzed using either a Beckman CytoFLEX or a Cytek DxP Athena flow cytometer. At least 100 000 single cells were analyzed per sample.

### 5.13 In Vivo PI Staining Assay

To label dead tumor cells in vivo, C57BL/6 mice with MB49‐derived subcutaneous tumor xenografts were intraperitoneally injected with 5 mg/kg propidium iodide (YEASEN, Cat. 40711ES60) 24 h after the final adenovirus treatment. Mice were euthanized 30 min post‐injection for tumor harvesting. Excised tumor tissues underwent immediate embedding in OCT (SAKURA, Cat. 4583), cryosectioning, and subsequent confocal microscopic analysis (ZEISS, LSM 800).

### 5.14 RealTime‐Glo Extracellular ATP Assay

Extracellular ATP release was quantified using the RealTime‐Glo Extracellular ATP Assay (Promega, Cat. No. GA5011) according to the manufacturer's instructions. For 5637 and BIU87 cells, 1 × 10^4^ cells were seeded into 96‐well plates and allowed to adhere for 12 h under standard culture conditions (37°C, 5% CO_2_). Cells were then transduced with adenoviral vectors encoding PyroRACS. At 36 h post‐transduction, pyroptosis was induced by 660‐nm light irradiation (1 mW cm^−^^2^, 30 s). Immediately after irradiation, the culture medium was replaced with fresh CO_2_‐independent complete medium supplemented with 10% fetal bovine serum (FBS), and luminescence was monitored at 37°C using a luminometer (VICTOR Nivo, PerkinElmer). Luminescence signals were recorded every 15 min for 24 h.

For HeLa cells, 5 × 10^5^ cells were seeded into 12‐well plates and cultured for 12 h before transfection with 1 µg of the PyroRACS expression plasmid using Lipofectamine 3000 (Thermo Fisher Scientific). At 24 h post‐transfection, pyroptosis was induced by 660‐nm light irradiation (1 mW cm^−^^2^, 30 s). Culture supernatants were collected at the indicated time points after irradiation, and extracellular ATP levels were determined using the RealTime‐Glo Extracellular ATP Assay according to the manufacturer's instructions.

### 5.15 LDH Release Assay

Cytotoxic lactate dehydrogenase (LDH) release dynamics were quantified using a commercial LDH assay kit (MCE, Cat. HY‐K1090) according to the manufacturer's instructions. Briefly, 5 × 10^4^ 5637 or BIU87 cells/well were plated in 96‐well plates. After 12 h adhesion, cells were infected with adenoviruses expressing PyroRACS. At 36 h post‐transduction, medium was replaced with complete medium containing 2% FBS to minimize serum‐derived LDH interference. Pyroptosis was triggered by 660‐nm photostimulation (1 mW/cm^2^, 30 s). At 6, 12, 24 h post‐induction, 50 µL supernatant was added to an equal volume of working solution, incubated at RT for 30 min, followed by adding 50 µL of stop solution. The absorbance at 490 nm was immediately measured using a microplate reader. Additionally, at the detection endpoint, 10 µL lysis solution was added to control wells to determine the maximum LDH release. The dynamic release of LDH was evaluated by the ratio of absorbance values at each time point to the absorbance value at maximum LDH release. LDH release (%) = [(Sample A490 – Background) / (Maximal LDH A490 – Background)] × 100

### 5.16 Enzyme‐Linked Immunosorbent Assay (ELISA)

The levels of IL‐18 and HMGB1 in cell culture supernatants were measured using the IL‐18 ELISA kit (TechiSun, Cat. TQ‐KL‐EL2143H) and HMGB1 ELISA kit (ELGBIO, Cat. EWE1681Hu) according to the manufacturers’ instructions. Briefly, culture supernatants were collected at designated time points and centrifuged at 2000 × g for 10 min at 4°C. The clarified supernatants were transferred to new Eppendorf tubes and supplemented with protease inhibitors at a 1:100 ratio. Subsequently, the samples were added to ELISA plates pre‐coated with capture antibodies and incubated at 37°C for 90 min. After three washes, biotin‐conjugated detection antibodies were added and incubated at 37°C for 50 min. Following three additional washes, HRP‐conjugated reagents were applied and incubated at 37°C for 50 min. Plates were then washed five times before adding TMB substrate, followed by incubation at 37°C for 20 min. The reaction was terminated with stop solution, and absorbance was immediately measured at 450 nm.

### 5.17 Hydrodynamic Tail Vein Injection

To evaluate the in vivo performance of distinct light‐activated Cre recombinase systems, plasmid DNA encoding optogenetic constructs and luciferase reporter (120 µg) were mixed at a 1:1:1 mass ratio in Ringer's solution. The injection volume of Ringer's solution per mouse (mL) = body weight (g) / 10 (g/mL). Using a 3 mL syringe fitted with a 27‐gauge needle, the plasmid mixture was injected intravenously via the tail vein into 8‐week‐old male C57BL/6 mice. The injection was completed rapidly within 5 s. At 8 h post‐injection (hpi), mice underwent specific light illumination to induce luciferase expression (Table S3), while control mice remained in darkness. Bioluminescence imaging was performed at 24 hpi to assess reporter gene expression.

### 5.18 Bioluminescence Imaging

Prior to bioluminescence imaging, D‐luciferin potassium salt (YEASEN, Cat. 115144) was intraperitoneally administered at 150 mg/kg as the luminescent substrate. This injection was performed 10 min before image acquisition. Mice were anesthetized using isoflurane (RWD, Cat. R5102210) and maintained under anesthesia throughout the procedure. Dynamic imaging commenced at 8 min post‐luciferin injection and continued until the bioluminescent signal reached peak intensity.

### 5.19 Animal Study

Animal experiments were approved by the Experimental Animal Welfare and Ethics Committee of China Science Industry Holdings (Shenzhen) Co., Ltd (No. 20240068). C57BL/6 and BALB/c nude mice were purchased from Shenzhen TopBiotech Co., Ltd.

To validate RACS‐mediated transcriptional activation in vivo, MB49 reporter cells stably expressing RACS and luciferase (5 × 10^6^ cells/mouse) were engrafted into BALB/c nude mice via subcutaneous implantation or tail vein injection to establish site‐specific tumor models. Optogenetic induction commenced 7 days post‐engraftment, beginning with intraperitoneal administration of PCB (10 mg/kg) 30 min prior to illumination. Mice received 660‐nm photostimulation (15 mW/cm^2^, 30 min) via calibrated LED arrays while freely ambulating in a custom transparent chamber. Bioluminescence imaging (IVIS Spectrum) was performed at 48 h post‐illumination. Signal intensity was quantified using Living Image software.

To evaluate the therapeutic efficacy and biosafety of controllable pyroptosis, a syngeneic bladder cancer model was established by subcutaneously injecting 1 × 10^6^ MB49 cells into 8‐week‐old C57BL/6 mice. Five days after tumor inoculation, 10 µL of adenoviral vectors (1 × 10^9^ PFU) co‐encoding PyroRACS were administered via intratumoral injection. At 48 h post‐injection, pyroptosis was spatially induced by focal irradiation with a custom‐designed 660 nm laser setup (15 mW/cm^2^, 5 min; Figure S17C). The treatment was repeated twice per week for a total of four treatment cycles. Body weight and tumor volume were measured every 48 or 72 h. Tumor volume was calculated as length × width^2^/ 2. At the end of the treatment, major organs, including the heart, liver, spleen, and kidneys, were harvested for hematoxylin and eosin (H&E) staining, and peripheral blood was collected for serum biochemical analysis to evaluate the biosafety of the treatment. Histological examination and serum biochemical analyses were performed by Servicebio (Wuhan, China).

### 5.20 Tumor Infiltrating Immune Cell Analysis

Tumor specimens were dissected from mice and cleared of surface adipose and connective tissues, followed by mincing into 2–4 mm^3^ fragments using scissors. Single‐cell suspensions were prepared using a mouse tumor dissociation kit (Miltenyi Biotec, Cat. 130096730) in accordance with the manufacturer's protocol, incorporating mechanical disruption via gentleMACS Dissociator (Miltenyi Biotec) and enzymatic digestion at 37°C for 40 min. The resulting suspension was filtered through a 70‐µm cell strainer and centrifuged at 300 ×g for 5 min. Erythrocytes were lysed using RBC lysis buffer (eBioscience, Cat. 00433357) for 5 min at RT, followed by two washes. Cell viability was assessed by staining with Fixable Viability Stain 510 (BD Biosciences, 1:1000 dilution, 15 min incubation at RT in the dark). Prior to surface staining, Fc receptors were blocked by incubating cells with 2 µL anti‐mouse CD16/32 antibody (Biolegend, Cat. 101320) per 100 µL cell suspension for 10 min at 4°C. Surface marker staining was performed by incubating cells with antibody cocktails for 30 min at 4°C protected from light. After two washes, intracellular staining was conducted using the Cytofix/Cytoperm Fixation/Permeabilization Kit (BD Bioscience, Cat. 554714): cells were fixed/permeabilized for 20 min at 4°C, washed twice with Perm/Wash buffer, and incubated with intracellular antibodies for 45 min at 4°C. Finally, cells were resuspended in 500 µL staining buffer (PBS containing 2% bovine serum albumin) and analyzed within 24 h using a DxP Athena flow cytometer (Cytek Biosciences). Antibodies were purchased from BioLegend unless specified and listed as below: APC‐Cy7 anti‐CD45 (Cat. 103116), PE anti‐CD3 (Cat. 100206), FITC anti‐CD8 (Cat. 100706), BV711 anti‐CD4 (Cat. 100550), APC anti‐F4/80 (Cat. 123116), BV421 anti‐CD86 (Cat. 105032), and BV785 anti‐CD206 (Cat. 141729); PE anti‐CD11b (BD Biosciences, Cat. 557397).

### 5.21 Statistical Analysis

Statistical analyses were performed using GraphPad Prism 10. Unless otherwise specified, intergroup differences were assessed by unpaired *t*‐test, with p < 0.05 considered statistically significant. Generally, data are presented as the mean ± standard deviation (SD), with at least three biological replicates per group. The general statistical workflow was as follows: data normality was first evaluated using the Shapiro–Wilk test together with Q–Q plot analysis. After confirming that the data followed, or approximately followed, a normal distribution, intergroup comparisons were performed using an unpaired *t*‐test. For data with equal variances, Student's *t*‐test was used, whereas Welch's *t*‐test was applied when variances were unequal.

## Author Contributions

W.H. conceived the study. M.Z. and W.H. designed this project. Experimental work was performed by M.Z. and Y.M. Data were analyzed by M.Z., Z.L., Y.S., W.H., and W.C. M.Z. wrote the original manuscript draft. All authors reviewed, edited, and approved the final manuscript.

## Funding

This work was supported by the Guangdong Special Support Program (2021JC06Y578), the Shenzhen Medical Research Fund (B2402036), the Natural Science Foundation of China (82302933 and 82473168), the Shenzhen Portion of Shenzhen‐Hong Kong Science and Technology Innovation Cooperation Zone (HTHZQSWS‐KCCYB‐2023060), the Guangzhou Key R&D Program (2024B01J1211), the Guangdong Engineering Technology Research Center for Clinical Application of Cancer Genome (2023B191), the Research Fund from Synthetic Biology Research Center of Shenzhen University, and the Shenzhen High‐level Hospital Construction Fund.

## Conflicts of Interest

The authors declare no conflicts of interest.

## Supporting information




**Supporting File**: advs76768‐sup‐0001‐SuppMat.docx.

## Data Availability

All data are available in the main text, the supplementary materials, or from the corresponding author upon reasonable request.
