# Supplementary material for: Engineering a 660 nm‐Responsive Optogenetic Inducer of Pyroptosis for Precision Cancer Therapy
Source: Adv Sci (Weinh). 2026 Jul 31;13(58):e76768. doi: 10.1002/advs.76768 (PMC13427370; doi:10.1002/advs.76768)
Supplement: Supplementary file 1 [file ADVS-13-e76768-s001.docx]

Supporting Information

**Engineering a 660 nm-Responsive Optogenetic Inducer of Pyroptosis for Precision Cancer Therapy**

*Mengkai Zhang, Zhichao Li, Yonghao Ma, Yangyang Sun, Wei Chen, Weiren Huang**

This PDF file includes:

Figures S1 to S17

Tables S1 to S4

Supplementary Text

**Figure S1 Screening of gasdermin N-terminal domains for efficient pyroptosis induction.**

**A.** Experimental design for GSDM^NT^-mediated pyroptosis screening. Expression vectors encoding Gasdermin NT domains (GSDMA-GSDME) and EGFP (to label transfected cells) were transfected into 293T cells. Pyroptosis was analyzed in situ at 24 h post-transfection by Annexin V-FITC/PI staining. CMV, CMV promoter; pEF1α, EF1α promoter; pA, polyA signal. **B.** Fluorescence microscopy of pyroptotic cells. Representative images showing cell death induction by GSDM^NT^s. Arrowheads indicate viable EGFP^+^ transfected cells. Scale bar: 20 µm. *n* = 3 biological replicates. BF, bright field. PI, propidium iodide.

**Figure S2 Uncontrolled pyroptosis triggered by core promoter basal leakage, related to Figure 1.**

**A.** Pyroptosis induction through REDMAP-mediated GSDMD^NT^ expression. 293T cells were transfected with plasmids encoding PhyA-Gal4, FHY1-VP64, GSDMD^NT^, or an empty vector control. Cells were cultured in the dark for 24 h, followed by in situ Annexin V-FITC/PI staining and fluorescence imaging. PhyA, phytochrome A; FHY1, far-red elongated hypocotyl 1; Gal4, Gal4 DNA-binding domain; VP64, a tetrameric VP16 transactivation domain; UAS, upstream activating sequence; miniCMV, minimal CMV promoter; pA, polyA signal. **B.** Uncontrolled pyroptosis caused by REDMAP leakage. Representative images of Annexin V/PI staining showing pyroptotic cells resulting from basal REDMAP activity. Cells transfected with the empty vector served as the negative control (NC). Scale bar, 20 μm. *n* = 3 biological replicates. **C.** Experimental design for pyroptosis induction via Tet-On-mediated GSDMD^NT^ expression. 293T cells transfected with plasmids encoding Tet-On and GSDMD^NT^ were cultured for 24 h in doxycycline-free medium before Annexin V/PI staining and imaging. TetO, tet operator; pPGK, PGK promoter; rtTA, reverse tetracycline-controlled transactivator. **D.** Tet-On leakage-induced pyroptosis. Fluorescence images of Annexin V^+^/PI^+^ cells. Scale bar: 20 μm (*n* = 3 biological replicates). Dox, doxycycline**. E.** Experimental workflow for assessing pyroptosis mediated by basal activity of the minimal CMV promoter. In the vector design, GSDMD^NT^ expression was driven by a minimal CMV promoter (miniCMV), with co-expressed enhanced green fluorescent protein (EGFP) serving as a transfection marker. At 24 h post-transfection, cells were stained with Annexin V-AF647/PI, followed by fluorescence microscopy analysis. **F.** Confocal imaging of miniCMV leakage-induced pyroptosis. Scale bar: 20 μm. **G.** Minimal promoter activity comparison. 293T cells were transfected with plasmids encoding a firefly luciferase reporter driven by distinct minimal promoters. The bioorthogonal 5xUAS element was positioned upstream of the minimal promoter (miniPro) to insulate endogenous regulatory signals. Luciferase activity was measured 48 h post-transfection. Data are presented as mean ± SD, *n* = 3 biological replicates. miniYB, minimal YB promoter; miniTK, minimal TK promoter. **H**. Pyroptosis triggered by miniYB promoter basal activity. 293T cells were transfected with plasmids co-expressing GSDMD^NT^ (driven by miniYB promoter) and EGFP. In situ Annexin V/PI staining was performed 24 h post-transfection, followed by fluorescence microscopy analysis. Representative images show miniYB leakage-induced pyroptosis. Scale bar: 20 µm.

**Figure S3** **Negligible dark-state interaction between PhyA and FHY1 in REDMAP, related to Figure 1.**

**A.** Schematic of REDMAP optogenetic system for luciferase regulation. Upon 660-nm illumination in the presence of phycocyanobilin (PCB), FHY1-VP64 heterodimerizes with PhyA-Gal4, triggering nuclear translocation. The complex binds to the synthetic 5×UAS-miniCMV promoter to drive luciferase expression. Luc, luciferase. **B.** Experimental design for assessing REDMAP performance. REDMAP components (PhyA-Gal4, FHY1-VP64, and luciferase reporter) were expressed from three independent plasmids. Twenty-four hours post-transfection, 293T cells were either exposed to 660-nm light (1 mW/cm², 1 min) to induce gene expression or kept in the dark. Luciferase activity was quantified at 24 h post-transfection. **C.** Luciferase assay shows negligible interaction between PhyA and FHY1 in the dark. Data are presented as mean ± SD, *n* = 3 biological replicates, unpaired *t*-test. ns, not significant.

**Figure S4 Optimization of PCB for robust RACS performance, related to Figure 1.**

**A.** Workflow for PCB concentration screening. 293T cells co-transfected with RACS and luciferase reporter plasmids were treated with varying PCB concentrations (0-10 μM) at 24 h post-transfection. After a 30-min incubation, cells were illuminated (660 nm, 1 mW/cm², 1 min). Luciferase activity was measured at 48 h post-illumination. **B.** Effects of PCB concentration on RACS activity. Data are presented as mean ± SD. Unpaired *t*-test; *n* = 3 biological replicates; ns, not significant; **** p* < 0.001. **C.** Setup of PCB incubation duration prior to illumination. Cells were treated with 10 μM PCB at 24 h post-transfection, incubated for indicated durations, then immediately illuminated (660 nm, 1 mW/cm², 1 min). **D.** Effects of PCB incubation duration on RACS activity. Luciferase activity was analyzed at 48 h post-illumination. Data are presented as mean ± SD; *n* = 3 biological replicates; one-way ANOVA. ns, **** p* < 0.001. **E.** Schematic of PCB removal after illumination. Following a 30-min PCB incubation, transfected 293T cells were illuminated with 660-nm light (1 mW/cm², 1 min). The medium was replaced with fresh PCB-free complete medium either immediately or 6 h post-illumination. Luciferase activity was measured 48 h post-illumination. **F.** Effects of PCB removal on RACS performance. Data are presented as mean ± SD; two-tailed unpaired *t*-test, *n* = 4 biological replicates.

**Figure S5 Optimization of illumination parameters for RACS activation, related to Figure 1.**

**A.** Schematic representation of the illumination duration setup. **B.** Impact of illumination duration on RACS performance. 293T cells were exposed to light (660 nm, 1 mW/cm^2^) for the indicated durations. Luciferase activity was measured 48 h post-illumination. Data are presented as mean ± SD, *n* = 4 biological replicates. One-way ANOVA with Tukey's multiple comparisons test, ***** p* < 0.0001. **C.** Schematic diagram of multi-round illumination. 293T cells were treated with 10 μM PCB at 24 h post-transfection. Cells were illuminated (660 nm, 1 mW/cm², 30 s) at 30 min post-PCB addition; alternatively, additional illumination was applied at 6 or 12 h after PCB addition. **D.** Effect of illumination rounds on RACS performance. Data are presented as mean ± SD, *n* = 4 biological replicates. **E.** Schematic of pulsed illumination. Transfected 293T cells were subjected to pulsed illumination (660 nm, 1 mW/cm²) with a cycle pattern of 15 min on/15 min off for the specified durations. **F**. Effect of pulsed illumination duration on RACS performance. Luciferase activity was measured 48 h post-illumination. Data are presented as mean ± SD; *n* = 4 biological replicates. **G**. Effect of light intensity on RACS performance. Different light intensities were generated by adjusting the input voltage of the LED light source and calibrated using an illuminometer. Luciferase activity was analyzed 48 h post-illumination. Data are presented as mean ± SD, *n* = 3 biological replicates. One-way ANOVA with Tukey's multiple comparisons test, ***** p* < 0.0001.

**Figure S6 RACS performance in distinct mammalian cell lines, related to Figure 2.**

Plasmids encoding RACS and luciferase were transiently transfected into T24 (A) or N2A (B) cells. Twenty-four hours post-transfection, cells were incubated with 10 μM PCB and subsequently exposed to light (660 nm, 1 mW/cm^2^, 30 s) to induce luciferase expression. Luciferase activity was measured at 24 h post-illumination. Data are shown as mean ± SD; unpaired *t*-test, *n* = 3 (T24) or 4 (N2A) biological replicates.

**Figure S7 RACS-mediated luciferase expression in the subcutaneous tumor model, related to Figure 2.**

**A.** Bioluminescence images of subcutaneous tumors. Stably transduced MB49 cells expressing RACS and luciferase were subcutaneously inoculated into BALB/c nude mice. At 7 days post-inoculation, mice were illuminated with 660-nm light (15 mW/cm², 30 min) or kept in the dark. Bioluminescence imaging (IVIS Spectrum, PerkinElmer) was performed at 48 h post-illumination. Circles outline the regions of interest used for total flux analysis. **B.** Quantification of bioluminescence signals in mice (A). Total flux represents the integrated bioluminescence intensity within the defined region of interest. Data are presented as mean ± SD; unpaired *t*-test, *n* = 5 biological replicates. **C.** Bioluminescence images of isolated tumors.

**Figure S8 Dose titration of 4-hydroxytamoxifen for CreERT2 activation, related to Figure 3.**

A. Fluorescence imaging of mCherry-positive 293T cells. 293T cells co-transfected with CreERT2 and mCherry plasmids were exposed to the indicated concentrations of 4-hydroxytamoxifen at 24 h post-transfection. Fluorescence microscopy of mCherry^+^ cells was performed at 48 h post-treatment. Representative images from four biological replicates are shown. Scale bar: 50 µm. **B.** Quantification of mean fluorescence intensity (MFI) in mCherry-positive 293T cells. MFI of mCherry^+^ cells was quantified using ImageJ. Data are presented as mean ± SD. One-way ANOVA followed by Tukey's multiple-comparison test. *n* = 4 biological replicates. ns, not significant.

**Figure S9 Transfection efficiency-normalized comparison of EGFP^+^ cells between RACS and CreERT2, related to Figure 3.**

To assess transfection efficiency, 293T cells were transfected with equal amounts of an EGFP reporter plasmid. The proportions of EGFP-positive cells under basal (**A**) and induced (**B**) conditions were normalized to transfection efficiency prior to comparative analysis. Data are presented as mean ± SD. Unpaired *t*-test; *n* = 3 biological replicates. ns, not significant; ** *p* < 0.01.

**Figure S10 In vivo DNA recombination mediated by FISC, related to Figure 3.**

**A.** Assessment of FISC activity via bioluminescence imaging. Following hydrodynamic delivery of plasmids encoding FISC and luciferase reporter, mice were subjected to pulsed 730-nm illumination (20 mW/cm²; 15 min on/off cycles) for 16 h, starting 8 h post-injection. Bioluminescence imaging was performed at 16 h post-illumination. Circles outline the regions of interest (ROIs) used for total flux analysis. **B.** Quantification of bioluminescence signals in mice (A). Total flux represents the bioluminescence intensity within the defined region of interest. Data are presented as mean ± SD. Unpaired *t*-test; *n* = 4 biological replicates. ns, not significant.

**Figure S11 Western blot analysis of cell death-associated molecules, related to Figure 4**.
**A**. Western blot analysis of key marker molecules associated with different cell death pathways. GAPDH was used as a loading control. The images shown are representative of three independent experiments. **B**. Full membrane images of the Western blots shown in Figure 4I. **C**. Western blot analysis of MLKL phosphorylation in HeLa cells. HeLa cells were collected 24 h after light-induced pyroptosis to assess MLKL phosphorylation levels. HeLa cells treated with TSZ (TNF-α, 20 ng/mL; birinapant, 1 μM; z-VAD-FMK, 20 μM) were used as the positive control, and non-irradiated cells served as the negative control.

**Figure S12 Analysis of pyroptosis-associated DAMP release and cytokine secretion, related to Figure 4**.

**A**. Flow cytometric analysis of CRT-positive HeLa cells. HeLa cells were transfected with plasmids expressing PyroRACS for 24 h, followed by light irradiation (660 nm, 1 mW/cm², 30 s) to induce pyroptosis (ON). Non-irradiated cells served as the negative control (OFF). Twelve hours after light irradiation, cells were harvested and analyzed by flow cytometry for cell-surface calreticulin (CRT) exposure. **B**. Quantification of CRT-positive HeLa cells. CRT-positive/7-AAD-negative cells were defined as cells exhibiting cell-surface exposure of CRT. Data are presented as mean ± SD; multiple unpaired *t*-tests; *n* = 4 biological replicates. 7-AAD, 7-aminoactinomycin D. **C** and **D**. Time-course analysis of LDH and IL-18 release. Cell culture supernatants were collected at 6, 12, and 24 h after light irradiation, and the levels of LDH (C) and IL-18 (D) were quantified. Data are presented as mean ± SD. Multiple unpaired *t*-tests; *n* = 4 biological replicates; ns, not significant; * *p* < 0.05; *** *p* < 0.001; **** *p* < 0.0001. LDH, lactate dehydrogenase; IL-18, interleukin-18.

**Figure S13 Optogenetic control of apoptosis via RACS, related to Figure 4.**

A. Experimental workflow for controllable apoptosis induction. 293T cells co-transfected with RACS and rCaspase-3 (rCASP3) plasmids were illuminated (660 nm, 1 mW/cm², 30 s) at 24 h post-transfection to activate rCaspase-3-mediated apoptosis. Apoptotic cells were analyzed at 18 h post-illumination by Annexin V-FITC/PI staining and fluorescence microscopy. **B**. Fluorescence imaging of apoptotic cells. Representative images showing Annexin V^+^/PI^-^ cells (early apoptosis, white arrowheads) and Annexin V^+^/PI^+^ cells (late apoptosis). Scale bar: 20 µm (*n* = 2 biological replicates).

**Figure S14 Assessment of background cytotoxicity of PyroRACS, related to Figure 5.**
**A**. Dynamic analysis of cell viability in 5637 cells. 5637 cells were infected with an adenovirus expressing PyroRACS and either exposed to irradiation every 24 h (660 nm, 1 mW/cm², 30 s), treated with 5 μM PCB at 12 h post-infection, or subjected to combined light irradiation and PCB treatment at 48 h post-infection to induce pyroptosis. Cell viability was measured using the CellTiter-Glo assay at 12, 24, 48, and 72 h after infection. Cells infected with the empty-vector adenovirus served as the negative control. Data are presented as mean ± SD. Two-way ANOVA followed by Tukey’s multiple-comparison test. *n* = 4 biological replicates. **B**. Western blot analysis of GSDMD^NT^ expression. 5637 cells were infected with Ad-PyroRACS for 24 h and subsequently cultured in the dark in medium containing 5 μM PCB until 72 h. Flag-tagged GSDMD^NT^ expression was detected by Western blotting at 72 h. Cells subjected to light-induced pyroptosis for 24 h were used as the positive control, and empty-vector-infected cells served as the negative control. **C** and **D**. Evaluation of the basal cytotoxicity of PyroRACS in mouse neural stem cells (C17.2). Cytotoxicity was assessed by measuring cell viability (C) and LDH release (D). C17.2 cells were infected with Ad-PyroRACS (MOI = 100), and cell viability was measured using the CellTiter-Glo assay at 24, 48, and 72 h post-infection. Following infection, cells were either continuously cultured for up to 72 h or exposed to 660-nm light irradiation (1 mW cm⁻², 30 s) at 48 h to induce cell death. Cells infected with the empty-vector adenovirus served as the negative control (NC). Data are presented as mean ± SD. One-way ANOVA followed by Tukey's multiple-comparison test. *n* = 4 biological replicates. For LDH quantification (D), culture supernatants collected at 72 h were analyzed. Data are presented as mean ± SD. Welch ANOVA followed by Dunnett multiple-comparison test; *n* = 4 biological replicates. **E** and **F**. Evaluation of the basal cytotoxicity of PyroRACS in human kidney-2 (HK-2) cells. Experimental conditions were identical to those described for C17.2 cells, except that HK-2 cells were infected with Ad-PyroRACS at an MOI of 50. Cell viability (E) and LDH release (F) were assessed as described above. ** *p* < 0.01, *** *p* < 0.001, **** *p* < 0.0001.

**Figure S15 Optogenetic induction of pyroptosis via PyroRACS in MB49 cells, related to Figure 6.**

MB49 cells were transduced with adenoviruses encoding PyroRACS (MOI = 1000). At 36 h post-transduction, GSDMD^NT^ expression was activated by 660-nm illumination (1 mW/cm^2^, 30 s) to trigger pyroptosis. At 48 h post-illumination, pyroptotic cells were subjected to in situ Annexin V/PI staining and fluorescence imaging. Representative images from two biological replicates. Scale bar: 20 µm.

**Figure S16 Biosafety evaluation of PyroRACS-mediated tumor therapy in vivo, related to Figure 6.
A.** Monitoring of mouse body weight. Body weight was measured every 2–3 days. Data are presented as mean ± SD, *n* = 7 biological replicates. **B**. Blood chemistry analysis of mice. Peripheral blood was collected after the final adenoviral treatment, and serum AST and CREA levels were measured by blood biochemical analysis to assess potential liver and kidney injury, respectively. Data are presented as mean ± SD; unpaired *t*-test, *n* = 7 biological replicates. AST, aspartate aminotransferase; CREA, creatinine.

**Figure S17 Schematic of illumination patterns.**

For cell illumination (**A**), cells were cultured in customized cell culture plates, with independent LED sources positioned at the top of each well. For animal illumination, mice were placed in customized transparent chambers to allow free movement, with a customized 660-nm LED array installed at the bottom of the chamber (**B**). Alternatively, after anesthesia, the tumor was locally irradiated with a 660-nm laser (**C**). The light intensity of the LEDs was adjusted by changing the voltage.

**Table S1. Different linkers used in Figure 1E.**

| Linker ID | Amino acid sequence | Annotation |
| --- | --- | --- |
| L1 | ASGSGGGGDV | flexible |
| L2 | SDSAGSAGSAGSGSG | flexible |
| L3 | GGGGSGGGGSGGGGR | flexible |
| L4 | LEASPSNPGA | semi-flexible |
| L5 | LEASPSNPGASN | semi-flexible |
| L6 | LEASPSNPGASNGS | semi-flexible |
| L7 | LEASPSNPGASNGSGT | semi-flexible |
| L8 | APTLADLAVDLAALRPLEHPNPPLQRAAEALL | long |
| L9 | SGGSSGGSSGSETPGTSESATPESSGGSSGGS | long |
| L10 | EAAAKEAAAKEAAAK | rigid |

**Table S2. Nucleotide sequences of loxP and its variants.**

| Name | Nucleotide sequence |
| --- | --- |
| loxP | ATAACTTCGTATA - GCATACAT - TATACGAAGTTAT |
| lox2272 | ATAACTTCGTATA - GGATACTT - TATACGAAGTTAT |
| lox511 | ATAACTTCGTATA - GTATACAT - TATACGAAGTTAT |
| lox66 | ATAACTTCGTATA - GCATACAT - TATACGAACGGTA |
| lox71 | TACCGTTCGTATA - GCATACAT - TATACGAAGTTAT |

Note: loxP and lox511 were used to construct double-floxed open reading frame.

**Table S3. Illumination parameters for optogenetic activation.**

| **Optogenetic**  **tools** | **Wavelength (nm)** | **Intensity**  **(mW/cm²)** | **Duration** | **Pulse cycle** | **Platform** | **Reference** |
| --- | --- | --- | --- | --- | --- | --- |
| **PACre** | 450 | 5 | 30 s | Continuous | *In vitro* | [33] |
| **RACS** | 660 | 1 | 30 s | Continuous | *In vitro* | This study |
| **RedPACre** | 660 | 1 | 24 h | 15-min on/off | *In vitro* | [34] |
| **FISC** | 730 | 5 | 12 h/day for 2 days | 15-min on/off | *In vitro* | [36] |
| **PACre** | 450 | 20 | 16 h | 15-min on/off | *In vivo* | [33] |
| **RACS** | 660 | 15 | 5 (rest) or 30 (motion) min | Continuous | *In vivo* | This study |
| **RedPACre** | 660 | 10 | 16 h | 15-min on/off | *In vivo* | [34] |
| **FISC** | 730 | 20 | 16 h | 15-min on/off | *In vivo* | [36] |

**Table S4. Details of plasmids used in this study.**

| Plasmid | Description | Used in | |
| --- | --- | --- | --- |
| P1 | pCMV-GSDMA3^NT^-EF1α-EGFP-pA | Fig. S1 | |
| P2 | pCMV-GSDMB^NT^-EF1α-EGFP-pA | Fig. S1 | |
| P3 | pCMV-GSDMD^NT^-EF1α-EGFP-pA | Fig. S1 | |
| P4 | pCMV-GSDME^NT^-EF1α-EGFP-pA | Fig. S1 | |
| P5 | pCMV-PhyA-Gal4-pA | Fig. S2, S3 | |
| P6 | pCMV-FHY1-VP64-pA | Fig. S2, S3 | |
| P7 | 5XUAS-miniCMV-GSDMD^NT^-pA | Fig. S2 | |
| P8 | 7XTetO-miniCMV-GSDMD^NT^-pA-pPGK-rtTA-pA | Fig. S2 | |
| P9 | 5XUAS-miniCMV-GSDMD^NT^-pA-pEF1α-EGFP-pA | Fig. S2 | |
| P10 | 5XUAS-miniCMV-Firefly Luciferase-pA | Fig. S2,S3 | |
| P11 | 5XUAS-miniTATA-Firefly Luciferase-pA | Fig. S2 | |
| P12 | 5XUAS-miniTK-Firefly Luciferase-pA | Fig. S2 | |
| P13 | 5XUAS-miniYB-Firefly Luciferase-pA | Fig. S2 | |
| P14 | 5XUAS-miniYB-GSDMD^NT^-pA-pEF1α-EGFP-pA | Fig. S2 | |
| P15 | CMV-PhyACreN59-pA | Fig. 1b | |
| P16 | CMV-FHY1CreC60-pA | Fig. 1b | |
| P17 | CMV-CreC60FHY1-pA | Fig. 1b | |
| P18 | CMV-CreN59PhyA-pA | Fig. 1b | |
| P19 | CMV-PhyACreC60-pA | Fig. 1b | |
| P20 | CMV-FHY1CreN59-pA | Fig. 1b | |
| P21 | CMV-CreC60PhyA-pA | Fig. 1b | |
| P22 | CMV-CreN59FHY1-pA | Fig. 1b | |
| P23 | CMV-PhyACreN104-pA | Fig. 1b | |
| P24 | CMV-FHY1CreC106-pA | Fig. 1b | |
| P25 | CMV-CreN104PhyA-pA | Fig. 1b | |
| P26 | CMV-CreC106FHY1-pA | Fig. 1b | |
| P27 | CMV-PhyACreC106-pA | Fig. 1b | |
| P28 | CMV-FHY1CreN104-pA | Fig. 1b | |
| P29 | CMV-CreC106PhyA-pA | Fig. 1b | |
| P30 | CMV-CreN104FHY1-pA | Fig. 1b | |
| P31 | CMV-PhyA-Linker1-CreN59-pA | Fig. 1d, e | |
| P32 | CMV-PhyA-Linker2-CreN59-pA | Fig. 1d, e | |
| P33 | CMV-PhyA-Linker3-CreN59-pA | Fig. 1d, e | |
| P34 | CMV-PhyA-Linker4-CreN59-pA | Fig. 1d, e |  |
| P35 | CMV-PhyA-Linker5-CreN59-pA | Fig. 1d, e | |
| P36 | CMV-PhyA-Linker6-CreN59-pA | Fig. 1d, e | |
| P37 | CMV-PhyA-Linker7-CreN59-pA | Fig. 1d, e | |
| P38 | CMV-PhyA-Linker8-CreN59-pA | Fig. 1d, e | |
| P39 | CMV-PhyA-Linker9-CreN59-pA | Fig. 1d, e | |
| P40 | CMV-PhyA-Linker10-CreN59-pA | Fig. 1d, e | |
| P41 | CMV-FHY1-Linker1-CreC60-pA | Fig. 1d, e | |
| P42 | CMV-FHY1-Linker2-CreC60-pA | Fig. 1d, e | |
| P43 | CMV-FHY1-Linker3-CreC60-pA | Fig. 1d, e | |
| P44 | CMV-FHY1-Linker4-CreC60-pA | Fig. 1d, e | |
| P45 | CMV-FHY1-Linker5-CreC60-pA | Fig. 1d, e | |
| P46 | CMV-FHY1-Linker6-CreC60-pA | Fig. 1d, e | |
| P47 | CMV-FHY1-Linker7-CreC60-pA | Fig. 1d, e | |
| P48 | CMV-FHY1-Linker8-CreC60-pA | Fig. 1d, e | |
| P49 | CMV-FHY1-Linker9-CreC60-pA | Fig. 1d, e | |
| P50 | CMV-FHY1-Linker10-CreC60-pA | Fig. 1d, e | |
| P51 | CMV-loxP-Luciferase-loxP-pA | Fig. 1f, g | |
| P52 | CMV-lox511-Luciferase-lox511-pA | Fig. 1f, g | |
| P53 | CMV-lox2272-Luciferase-lox2272-pA | Fig. 1f, g | |
| P54 | CMV-lox66-Luciferase-lox66-pA | Fig. 1f, g | |
| P55 | CMV-lox71-Luciferase-lox71-pA | Fig. 1f, g | |
| P56 | CMV-lox511-loxP-Luciferase-lox511-loxP-pA | Fig. 1f, g |  |
| P57 | CMV-lox511-loxP-N-Flag-Luciferase-lox511-loxP-pA | Fig. 2e-g | |
| P58 | CMV-lox511-loxP-mCherry-lox511-loxP-pA | Fig. 2e-g | |
| P59 | 5XUAS-miniCMV-Luciferase-pA | Fig. 3a | |
| P60 | 5XUAS-miniYB-Luciferase-pA | Fig. 3a | |
| P61 | CMV-CreERT2-pA | Fig. 3b | |
| P62 | CMV-CreN59nMag-pA | Fig. 3g-i | |
| P63 | CMV-pMagCreC60-pA | Fig. 3g-i | |
| P64 | CMV-CreN104-Aff6_V18FΔN-pA | Fig. 3g-i | |
| P65 | CMV-DrBphCreC106-pA | Fig. 3g-i | |
| P66 | pA-BphS-YhjH-CMV-p65VP64-BldD-pA | Fig. 3g-i | |
| P67 | pA-CreN59Coh2-CMV-DIOLUC-pA | Fig. 3g-i | |
| P68 | pFRL-DocSCreC60-pA | Fig. 3g-i | |
| P69 | CMV-lox511-loxP-N-Flag-GSDMD^NT^-lox511-loxP-pA | Fig. 4 | |
| P70 | ITR-pA-DIOGSDMD^NT^-pCMV-pCBH-PhyACreN59-IRES-FHY1CreC60-pA-ITR | Fig. 5, 6 | |
| P71 | CMV-DIO-rCASP3-pA | Fig. S13 | |

Supplementary DNA Sequence Information

**RACS: PhyA-linker-CreN59**

gccaccATGGAGAAGAAGATGAGCGGATCTCGTCCCACACAGTCCAGCGAGGGATCTCGTAGATCTCGTCACTCCGCTCGTATCATCGCTCAGACCACCGTGGACGCCAAACTGCACGCCGATTTCGAGGAGAGCGGCTCCTCCTTTGATTACTCCACCAGCGTGAGGGTGACTGGTCCCGTGGTCGAGAACCAGCCTCCTAGGAGCGACAAGGTCACCACAACCTACCTCCATCATATCCAGAAGGGCAAGCTGATCCAGCCCTTTGGATGTTTACTGGCTTTAGACGAAAAGACCTTCAAGGTCATCGCCTACTCCGAGAACGCCTCCGAACTGCTCACAATGGCTTCCCACGCCGTGCCTAGCGTGGGAGAGCACCCCGTTCTGGGCATCGGCACCGATATCAGATCTTTATTTACCGCTCCCAGCGCTTCCGCTTTACAGAAGGCTCTCGGCTTCGGCGACGTGTCTTTACTGAACCCTATCCTCGTCCATTGTCGTACATCCGCCAAGCCCTTCTACGCCATTATCCATAGGGTGACCGGATCCATCATCATCGACTTCGAACCCGTTAAGCCCTATGAAGTGCCCATGACAGCCGCCGGAGCTTTACAGAGCTATAAGCTGGCCGCCAAGGCCATCACCAGACTCCAGTCTTTACCCAGCGGAAGCATGGAGAGGCTGTGCGACACCATGGTGCAAGAAGTCTTCGAGCTGACTGGTTACGATCGTGTCATGGCCTACAAGTTCCACGAGGACGACCACGGCGAGGTGGTGAGCGAAGTGACCAAGCCCGGTTTAGAACCCTATTTAGGTTTACACTATCCCGCCACAGACATCCCCCAAGCTGCCAGATTTTTATTTATGAAGAACAAGGTGAGAATGATCGTGGACTGCAACGCCAAGCACGCTAGGGTTTTACAAGATGAGAAGCTGAGCTTCGATTTAACCCTCTGCGGCAGCACTTTAAGAGCTCCCCACTCTTGTCATCTGCAATACATGGCCAATATGGACAGCATCGCTTCTTTAGTGATGGCCGTGGTGGTGAATGAGGAGGATGGAGAGGGCGATGCTCCCGATGCCACCACACAGCCTCAGAAGAGAAAGAGGCTGTGGGGTTTAGTGGTCTGCCACAACACCACCCCCAGATTTGTCCCTTTTCCCTTACGTTATGCTTGTGAATTTTTAGCCCAAGTGTTTGCCATCCACGTCAACAAGGAGGTCGAGCTGGACAACCAGATGGTGGAGAAGAACATTTTACGTACCCAGACACTCCTCTGCGACATGCTCATGAGGGACGCTCCCCTCGGCATCGTGAGCCAGTCCCCCAATATTATGGATTTAGTCAAGTGCGACGGCGCCGCCTTATTATACAAGGACAAGATCTGGAAGCTGGGCACCACCCCTAGCGAGTTCCATTTACAAGAAATCGCTTCTTGGCTGTGTGAGTACCACATGGATTCCACCGGTTTAAGCACCGATTCTTTACACGACGCTGGCTTTCCTAGGGCTTTATCTTTAGGCGACAGCGTCTGCGGAATGGCTGCCGTTCGTATCAGCAGCAAAGATATGATTTTCTGGTTTCGTTCCCATACCGCCGGCGAAGTGAGATGGGGCGGCGCCAAACACGACCCCGATGATAGGGACGATGCCAGAAGGATGCACCCCAGATCCTCCTTCAAGGCTTTTCTGGAGGTGGTGAAGACCAGAAGCTTACCTTGGAAGGACTACGAAATGGATGCCATCCACTCTTTACAGCTCATCTTACGTAACGCCTTTAAGGACAGCGAGACCACCGACGTGAACACAAAGGTGATCTATAGCAAGCTCAACGATCTCAAGATTGATGGAATCCAACTGGAGGCCTCCCCAAGCAACCCTGGCGCTATGTCTAACCTTCTGACTGTTCACCAGAATCTGCCTGCCTTGCCTGTGGACGCTACCTCTGATGAAGTACGGAAAAATCTCATGGATATGTTCAGGGACAGGCAAGCCTTCTCTGAGCATACGTGGAAAATGCTGCTGAGCGTTTGTCGGAGCTGGGCAGCCTGGTGTAAGCTGAATtaa

**RACS: FHY1-linker-CreC60**

gccaccATGGCCGAGGTGGAGGTCGACAACAACAACGAGAAGCCCAGCGAGATCAACAGCTTCCACCACATGATTATCAGCAGCAGCAAGAACGTGCTGAAGATGGAGGAGGTGGAGGTGTCCAAGAAGAGGAAGTTCCAGACCGACCAGAGCGACGAGCTGAGCTTATTACCTTTATCCAAACACACATGCTTTGCCAACGTGGCTTGTAGCGAGAACACCAACGGCAACAGCGAGATCGATACCGAGTACAGCATGTCCAGCTACGTGAACTCCACCACCTCCATGGAGTGCAACAACGACATCGAGATGAAGGAAGAATCCAGCGGCAGCTGCGGAGAGGACAAGATGATCAGCTTCGAGTCCCATTTAGACTATATCTACGGCACCCAGAATCTGGAGGACTTCTCCGAGAAGGTGATCGAGAACATTCTGTACCTCGACGAGCAAGAAGAGGAGGAGGAGGACGCCAAGGGCTGTAGCAGCAACGCTGCCAAGTTCGTGCTGTCCTCTGGTCGTTGGACCGTGAACCAAGATGATAGCACTTTACACGAAACCAAGAAGCCCACCATCGACCAAGAATTCGAGCAGTACTTTAGCACTTTAATGCTGCTGGAGGCCTCCCCAAGCAACCCTGGCGCTAACCGGAAGTGGTTCCCTGCAGAGCCAGAAGATGTGCGCGACTATCTGCTCTACCTGCAGGCTCGGGGGCTGGCTGTGAAGACCATCCAGCAGCACCTGGGGCAGCTCAACATGCTTCACAGAAGAAGCGGCCTGCCCAGACCATCGGACAGCAATGCTGTGAGCCTGGTGATGAGAAGAATTCGAAAGGAGAATGTGGATGCCGGAGAGAGGGCCAAGCAAGCCCTGGCCTTTGAGCGGACAGACTTTGACCAGGTGAGGAGCCTGATGGAGAACTCTGACAGGTGCCAGGACATTCGGAACCTCGCTTTCCTCGGCATAGCCTACAACACTCTCCTGCGGATCGCTGAGATCGCCAGGATCAGAGTCAAGGACATCTCGCGGACTGATGGAGGCAGGATGTTAATTCACATAGGCCGCACCAAGACACTGGTCAGCACAGCTGGCGTGGAAAAGGCGCTGTCCCTGGGTGTCACCAAACTGGTGGAGCGCTGGATCAGTGTGTCTGGCGTGGCAGATGACCCTAACAACTACCTCTTCTGCAGAGTGCGTAAAAATGGGGTGGCTGCCCCCTCAGCCACCAGCCAGCTGTCCACCCGGGCCTTAGAAGGGATCTTTGAAGCCACTCATAGGCTTATCTATGGAGCCAAAGATGATTCTGGCCAGCGCTACCTAGCATGGAGCGGCCACTCCGCCAGAGTGGGCGCGGCTAGAGACATGGCTAGAGCCGGAGTGAGCATCCCTGAAATCATGCAGGCAGGCGGGTGGACAAATGTCAACATCGTCATGAATTACATCCGCAACCTGGACAGTGAGACCGGCGCCATGGTGAGACTACTGGAGGACGGAGACtaa

**RACS: lox511-loxP-N-Flag-GSDMD^NT^-loxP-lox511**

ataacttcgtatagtatacattatacgaagttattctttgcaccattctaaagaataacagtgataatttctgggttaaggcaaataacttcgtatagcatacattatacgaagttatcctagcgccaccatgGATTACAAGGACGACGATGACAAGACCGGTATGGGGTCGGCCTTTGAGCGGGTAGTCCGGAGAGTGGTCCAGGAGCTGGACCATGGTGGGGAGTTCATCCCTGTGACCAGCCTGCAGAGCTCCACTGGCTTCCAGCCCTACTGCCTGGTGGTTAGGAAGCCCTCAAGCTCATGGTTCTGGAAACCCCGTTATAAGTGTGTCAACCTGTCTATCAAGGACATCCTGGAGCCGGATGCCGCGGAACCAGACGTGCAGCGTGGCAGGAGCTTCCACTTCTACGATGCCATGGATGGGCAGATACAGGGCAGCGTGGAGCTGGCAGCCCCAGGACAGGCAAAGATCGCAGGCGGGGCCGCGGTGTCTGACAGCTCCAGCACCTCAATGAATGTGTACTCGCTGAGTGTGGACCCTAACACCTGGCAGACTCTGCTCCATGAGAGGCACCTGCGGCAGCCAGAACACAAAGTCCTGCAGCAGCTGCGCAGCCGCGGGGACAACGTGTACGTGGTGACTGAGGTGCTGCAGACACAGAAGGAGGTGGAAGTCACGCGCACCCACAAGCGGGAGGGCTCGGGCCGGTTTTCCCTGCCCGGAGCCACGTGCTTGCAGGGTGAGGGCCAGGGCCATCTGAGCCAGAAGAAGACGGTCACCATCCCCTCAGGCAGCACCCTCGCATTCCGGGTGGCCCAGCTGGTTATTGACTCTGACTTGGACGTCCTTCTCTTCCCGGATAAGAAGCAGAGGACCTTCCAGCCACCCGCGACAGGCCACAAGCGTTCCACGAGCGAAGGCGCCTGGCCACAGCTGCCCTCTGGCCTCTCCATGATGAGGTGCCTCCACAACTTCCTGACAGATTAGggcgcgccataacttcgtataatgtatgctatacgaagttattaagaggtttcatattgctaatagcagctacaatccagctaccattctgcataacttcgtataatgtatactatacgaagttat

**FISC: p65-VP64-Linker-BldD**

gccaccATGCCAAGCGGTCAAATCTCTAACCAAGCGCTAGCCCTGGCCCCCTCCTCTGCCCCAGTCCTGGCTCAGACCATGGTGCCTTCCTCAGCCATGGTTCCCCTGGCCCAACCCCCTGCCCCCGCCCCTGTGCTCACCCCGGGCCCTCCCCAGAGCCTCATGGGGAGCGGAAGAGCTGACGCACTGGATGATTTTGATCTCGATATGCTGGGATCTGACGCCCTGGACGACTTCGACCTGGACATGCTGGGAAGTGACGCTCTGGATGATTTCGATTTGGACATGCTCGGCAGCGATGCCCTGGATGACTTTGACCTCGACATGCTCATTAATGCCAGTGGCTCTGGAGGCGGAGGTGATGTGATGGCTTCCCCCAAAAAGAAGAGAAAAGTGGAAGCTAGCAGCTCAGAGTACGCAAAGCAGCTGGGCGCGAAGCTGAGGGCCATCCGGACTCAGCAGGGCCTGAGCCTCCACGGCGTGGAGGAGAAGTCACAAGGCCGGTGGAAAGCCGTGGTGGTGGGGTCCTACGAACGGGGGGACAGAGCAGTGACAGTTCAGAGGCTGGCAGAGCTGGCAGACTTCTACGGGGTGCCTGTCCAGGAGCTGCTGCCTGGGACCACACCCGGCGGCGCGGCTGAGCCACCACCCAAATTGGTGCTGGACCTGGAGAGACTTGCCCACGTCCCACAGGAAAAGGCCGGGCCACTCCAGCGCTACGCTGCCACTATTCAGAGCCAAAGAGGTGACTATAACGGAAAGGTGCTGTCCATCCGGCAGGATGACCTGCGCACCTTGGCTGTCATCTATGACCAGTCTCCTTCTGTGCTCACAGAGCAGCTGATATCCTGGGGCGTCCTCGATGCTGACGCGAGGCGTGCCGTGGCACATGAAGAAAACtaa

**FISC: BphS-P2A-YhjH**

gccaccATGGCACGGGGCTGCCTGATGACCATTTCTGGAGGAACATTTGACCCCAGCATTTGTGAAATGGAGCCCATCGCAACCCCAGGCGCCATCCAGCCCCACGGGGCACTGATGACGGCCCGGGCCGACAGCGGAAGAGTGGCGCATGCCTCAGTCAACTTAGGTGAAATCCTCGGACTGCCAGCCGCAAGCGTGTTAGGCGCCCCAATCGGAGAAGTTATCGGCCGGGTGAATGAAATCCTGCTCCGGGAGGCCAGAAGGAGCGGTTCTGAAACCCCTGAGACCATCGGCTCATTCCGCAGAAGCGACGGCCAGCTGCTACACCTCCACGCATTCCAGTCTGGGGATTACATGTGCTTGGATATCGAACCTGTACGTGATGAAGACGGCAGACTCCCCCCTGGGGCCCGACAGAGCGTGATTGAGACATTCTCATCAGCCATGACTCAGGTGGAATTATGCGAGCTAGCCGTCCACGGCCTTCAGCTCGTGCTGGGTTACGATAGAGTCATGGCCTACCGCTTCGGAGCGGATGGCCATGGAGAGGTGATCGCCGAGCGGAGGAGACAAGACCTTGAGCCATATCTCGGTCTCCACTACCCAGCTTCTGACATACCACAGATCGCCAGAGCCCTGTACTTGAGGCAGAGGGTGGGCGCTATCGCAGATGCTTGCTACCGACCAGTGCCTCTCCTCGGCCATCCTGAATTGGATGACGGAAAACCTTTGGACCTGACACACTCCAGCCTGAGATCAGTATCCCCTGTTCACCTGGACTATATGCAAAACATGAATACAGCGGCCTCTCTTACTATCGGCCTGGCCGATGGAGACAGGTTGTGGGGAATGCTGGTGTGTCACAACACAACACCTCGCATTGCCGGCCCCGAGTGGAGAGCAGCGGCGGGCATGATTGGCCAAGTAGTGAGCCTGCTGCTTTCGAGACTAGGCGAGGTGGAAAATGCCGCCGAGACACTCGCTAGACAGAGCACGCTGTCTACCCTCGTGGAGCGTCTTAGCACAGGAGACACTCTCGCGGCAGCCTTCGTGGCTGCCGATCAGTTGATACTGGACCTGGTGGGCGCTTCCGCAGCCGTGGTGAGACTGGCAGGACAGGAACTTCACTTCGGCCGGACCCCCCCTGTGGATGCAATGCAGAAGGTCCTGGACTCTCTGGGAAGACCGTCCCCTTTGGAAGTGCTCAGTCTGGATGATGTCACACTTCGGCACCCAGAGTTGCCTGAGCTGTTGGCTGCAGGTTCCGGAATCCTGCTGCTGCCGCTGACCTCTGGTGATGGGGACCTCATCGCGTGGTTCAGACCTGAACATGTGCAAACCATCACATGGGGCGGCAACCCTGCAGAACACGGAACATGGAATCCAGCTACCCAGCGTATGAGGCCTCGCGCCAGCTTTGATGCCTGGAAGGAGACTGTTACCGGGCGCTCTCTCCCTTGGACATCCGCTGAGAGGAACTGTGCTCGGGAGCTGGGTGAGGCCATCGCCGCAGAGATGGCTCAGAGAACCCGGGCGGAGGAGCTGGAGCGCGTGGCTATGGTGGACAGCCTGACGCGCCTGTGGAACCGTCTGGGCATCGAAACCCTGCTGAAGCGGGAGTGGGAGTACGCCACCAGGAAGAACTCGCCAATCAGCATCGTGATGATCGACTTTGACAATTTTAAACAGATAAATGACCAGCACGGCCATCTGGTAGGAGATGAGGTTCTGCAGGGGAGCGCCCGGCTGATAATCAGCGTACTGGCCAGCTATGACATCCTGGGCCGCTGGGGAGGGGACGAGTTCATGCTGATCCTGCCTGGCTCCGGCAGAGAACAGACCGCCGTACTACTGGAAAGAATCCAGGCCACCATTGCACAGAACCCCGTGCCCACCTCCGCCGGCCCCATGGCCATTAGCCTTTCCATGGGTGGAGTCAGCGTCTTCACCAACCAGGGGGAGGCACTGCAGTACTGGGTCGAACAGGCAGACAACCAATTGATGAAAGTAAAGAGGCTGGGCAAGGGAAATTTTCAGCTGGCTGAATATCACCACCATCACCACCATGGCAGCGGCGCCACAAACTTCTCCCTGCTGAAGCAAGCAGGAGATGTCGAGGAGAACCCGGGTCCTAGCGGCATCCGTCAGGTCATCCAGAGAATTTCTAACCCTGAAGCCAGTATCGAAAGTTTGCAGGAGCGGAGATTCTGGCTGCAGTGTGAGCGGGCTTACACATGGCAGCCAATCTACCAGACCTGCGGGAGATTAATGGCTGTGGAGCTGCTGACTGTGGTGACTCACCCCCTGAACCCATCCCAGCGGTTGCCCCCGGATAGATACTTCACAGAGATCACGGTGAGTCACAGGATGGAAGTTGTGAAAGAGCAGATCGACCTACTGGCCCAGAAGGCCGACTTCTTCATCGAGCACGGCCTGCTCGCTAGCGTGAATATAGACGGCCCTACCCTCATCGCGCTGCGTCAGCAGCCTAAGATTCTCCGGCAGATCGAGAGGCTGCCCTGGCTTAGGTTTGAGCTGGTGGAGCACATCCGGCTGCCAAAAGATAGCACGTTCGCATCGATGTGTGAGTTTGGGCCCCTGTGGCTGGACGACTTTGGCACCGGCATGGCTAACTTCTCAGCCCTGTCCGAAGTGAGATATGATTACATCAAAATCGCGCGAGAGCTTTTCGTAATGCTGCGTCAGAGCCCCGAGGGCAGAACACTGTTCAGCCAGTTACTGCACCTCATGAACAGGTACTGCAGGGGCGTCATCGTAGAGGGAGTTGAAACTCCCGAAGAATGGAGGGATGTCCAGAACAGCCCAGCCTTTGCCGCCCAAGGTTGGTTTCTGTCGAGACCGGCCCCCATCGAGACACTCAACACCGCGGTGTTGGCCTTAtaa

**FISC: CreN(1-59)-L9-Coh2-NES**

gccaccATGAGTAACTTATTGACTGTTCACCAGAATCTGCCTGCGCTGCCTGTGGATGCCACATCTGATGAGGTGCGTAAGAACCTCATGGACATGTTCAGAGACAGGCAAGCCTTCTCTGAGCATACCTGGAAGATGCTGCTGTCCGTCTGCCGCAGCTGGGCAGCCTGGTGCAAACTTAACCTGGAAGCTAGTCCCAGCAACCCAGGGGCGAGCAATGGCAGCGTGGTGGTGGAAATCGGGAAGGTAACTGGCTCAGTGGGAACCACGGTGGAGATCCCTGTCTACTTCCGGGGCGTTCCTAGCAAGGGAATCGCCAACTGTGACTTTGTGTTCAGGTATGACCCCAACGTCCTAGAGATCATCGGCATTGACCCTGGAGACATCATCGTGGACCCAAACCCCACCAAAAGCTTTGACACAGCCATATACCCAGATAGAAAAATTATTGTCTTCCTTTTCGCAGAGGACTCTGGCACTGGCGCCTATGCCATCACCAAGGATGGTGTGTTTGCCAAGATCCGAGCCACTGTAAAGAGCAGTGCCCCGGGCTACATCACATTTGATGAAGTTGGAGGCTTTGCTGACAATGATCTGGTGGAGCAGAAAGTCAGCTTCATTGATGGTGGAGTGAATGTCTTCCTGCAGGTGAGAAAATACTCCCTGGATCTGGCTTCCCTCATCCTGTACGCTTACCAGCTCtaa

**FISC: pA-3×whiG-TATA**

cagacatgataagatacattgatgagtttggacaaaccacaactagaatgcagtgaaaaaaatgctttatttgtgaaatttgtgatgctattgctttatttgtaaccattataagctgcaataaacaagttaacaacaacaattgcattcattttatgtttcaggttcagggggaggtgtgggaggttttttaaacctcacgctacgctcactcacgctacgctcactcacgctacgctcacctgcaggagagggtatataatggaagctcgaattccagaagcttatactcagtgccctgactatatactcagtgccctgactat

**FISC: NLS-DocS-L9-CreC60**

gccaccATGGCATCCCCGAAGAAGAAAAGGAAGGTGGAGGCTTCCAGCACCAAACTATACGGAGACGTCAATGATGATGGCAAGGTCAACAGCACAGATGCTGTGGCCTTAAAGAGGTACGTGCTGCGGAGCGGCATAAGCATCAACACAGACAACGCTGACCTGAATGAAGATGGCCGGGTGAACTCTACGGACCTGGGAATCCTCAAGAGGTACATTTTGAAGGAAATAGATACCTTGCCCTACAAACTGGAAGCATCCCCTTCAAACCCTGGAGCCTCCAATGGCTCCAACCGGAAGTGGTTCCCTGCAGAGCCAGAAGATGTAAGAGACTACCTGCTCTACCTGCAAGCCCGGGGGCTCGCGGTGAAGACTATCCAGCAGCACCTGGGGCAACTCAACATGTTACACCGGAGAAGCGGCCTCCCTCGGCCATCAGACAGCAACGCTGTGTCCCTGGTGATGAGACGTATCAGAAAGGAAAATGTGGATGCCGGTGAGAGGGCCAAACAGGCCCTGGCCTTCGAGAGGACCGACTTTGACCAGGTGAGATCCCTCATGGAGAACAGTGACCGCTGCCAGGACATCAGAAATCTGGCTTTCCTGGGCATCGCTTACAATACCCTGCTGAGAATCGCAGAAATCGCCAGAATCAGGGTGAAAGATATCAGCAGAACGGATGGAGGCAGGATGCTGATTCACATCGGTCGCACAAAGACACTGGTGAGCACCGCTGGAGTGGAGAAGGCTCTATCTCTGGGGGTCACCAAGCTGGTGGAGCGCTGGATTTCAGTGAGCGGTGTGGCTGATGACCCCAACAACTATCTTTTCTGTAGAGTTAGAAAAAATGGCGTTGCCGCCCCCTCAGCTACTAGCCAGCTGAGCACCCGGGCCCTAGAAGGAATCTTTGAGGCTACCCATCGGCTCATCTATGGCGCGAAGGACGACAGCGGCCAGAGATACCTGGCCTGGAGTGGCCACTCTGCCCGGGTGGGCGCAGCCCGAGACATGGCCAGAGCAGGCGTCAGCATTCCAGAGATCATGCAGGCAGGGGGCTGGACAAACGTCAACATTGTCATGAACTACATCAGGAACTTGGACTCTGAGACCGGAGCCATGGTGCGTCTGCTGGAGGATGGAGACtaa

**RedCre: NLS-CreN104-Aff6_V18FΔN**

ATGGTGCCCAAGAAGAAGAGGAAAGTCGGCGGAACCTCTGATGAAGTCAGGAAGAACCTGATGGACATGTTCAGGGACAGGCAGGCCTTCTCTGAACACACCTGGAAGATGCTCCTGTCTGTGTGCAGATCCTGGGCTGCCTGGTGCAAGCTGAACAACAGGAAATGGTTCCCTGCTGAACCTGAGGATGTGAGGGACTACCTCCTGTACCTGCAAGCCAGAGGCCTGGCTGTGAAGACCATCCAACAGCACCTGGGCCAGCTCAACATGCTGCACAGGAGATCTGGCCTGGGTACCGCCAGCCCCTCTAACCCTGGAGCCTCCAACGGCAGCCTCGAGAAGTTTAACAAGGAGAAGTGGCAGGCTATTAAGGAGATTGCCTTCCTGCCTAACCTGAACCAGCCCCAGCACCTGGCTTTTATTGTGTCTCTGCTGGACGACCCTTCTCAGTCTGCTAACCTGCTGGCTGAGGCTAAGAAGCTGAACGACGCTCAGGCTCCTAAGtaa

**RedCre: NLS-DrBphP-CreC106**

ATGGTGCCCAAGAAGAAGAGGAAAGTCGGCGGAATGAGCCGCGACCCCCTGCCATTCTTCCCCCCCCTGTACCTGGGAGGACCCGAGATTACCACCGAAAACTGTGAACGAGAGCCTATCCACATCCCCGGAAGTATTCAGCCTCATGGCGCCCTGCTGACTGCTGATGGCCACAGTGGGGAAGTGCTGCAGATGTCACTGAACGCCGCTACCTTCCTGGGGCAGGAGCCTACTGTGCTGAGGGGACAGACCCTGGCAGCACTGCTGCCTGAACAGTGGCCAGCACTGCAGGCTGCACTGCCACCTGGCTGCCCCGATGCCCTGCAGTACAGGGCTACTCTGGACTGGCCTGCAGCTGGACACCTGAGCCTGACCGTCCATCGCGTGGGGGAACTGCTGATCCTGGAGTTCGAACCAACAGAGGCTTGGGATAGCACTGGCCCCCACGCACTGCGGAACGCTATGTTTGCACTGGAGTCCGCCCCCAATCTGAGAGCACTGGCCGAAGTCGCCACTCAGACCGTGCGGGAGCTGACAGGGTTCGATAGAGTCATGCTGTACAAGTTTGCCCCTGACGCTACTGGAGAAGTGATCGCTGAAGCACGGAGAGAGGGGCTGCACGCTTTCCTGGGACATCGATTTCCAGCCTCCGACATTCCAGCACAGGCTCGGGCACTGTATACCAGGCACCTGCTGCGACTGACAGCAGATACTCGCGCAGCAGCTGTCCCACTGGACCCCGTGCTGAACCCTCAGACCAATGCACCAACACCTCTGGGAGGAGCAGTGCTGAGAGCAACCTCTCCAATGCATATGCAGTATCTGAGGAATATGGGAGTCGGCAGCTCCCTGTCTGTGAGTGTGGTCGTGGGAGGACAGCTGTGGGGACTGATTGCCTGCCACCATCAGACACCCTACGTGCTGCCACCCGATCTGAGGACCACACTGGAGTATCTGGGGCGCCTGCTGTCTCTGCAGGTCCAGGTGAAAGAAGCAGCCGACGTCGCTGCATTCCGACAGAGTCTGCGAGAGCACCATGCACGGGTGGCCCTGGCCGCTGCACACTCACTGAGCCCTCATGACACACTGAGCGATCCAGCTCTGGACCTGCTGGGACTGATGAGAGCAGGAGGACTGATCCTGAGATTTGAAGGCAGGTGGCAGACTCTGGGAGAGGTCCCTCCAGCACCAGCTGTGGACGCTCTGCTGGCATGGCTGGAGACCCAGCCAGGAGCTCTGGTGCAGACAGATGCACTGGGACAGCTGTGGCCAGCAGGAGCAGACCTGGCTCCTTCAGCAGCTGGACTGCTGGCCATTTCCGTCGGAGAAGGATGGTCTGAGTGTCTGGTGTGGCTGCGCCCAGAACTGCGACTGGAGGTGGCTTGGGGAGGAGCAACCCCTGATCAGGCAAAGGACGATCTGGGACCACGCCACAGCTTTGACACATACCTGGAGGAAAAACGAGGCTATGCCGAGCCCTGGCACCCAGGGGAAATTGAGGAGGCACAGGATCTGAGAGACACACTGACAGGGGCACTGGGGGAGAGACTGTCCGTGATTCGCGATCTCAACCGGGCTCTGACCCAGTCCAATGCCGAGTGGCGTCAGTATGGCTTCGTCATCAGTCACCACATGCAGGAACCCGTCAGACTGATCTCCCAGTTTGCCGAGCTTCTGACTCGCCAACCCAGAGCCCAGGATGGCAGCCCGGACTCTCCCCAGACAGAGCGTATCACCGGCTTTCTGCTCAGGGAAACCTCACGACTCCGCTCTCTGACCCAGGACTTGCACACGTACACTGCGCTGCTGTCAGCACCTCCTCCAGTTAGGAGGCCAACACCACTTGGGCGAGTTGTGGACGATGTGCTGCAGGACCTCGAACCACGGATTGCCGATACAGGGGCCTCCATAGAGGTAGCTCCCGAACTGCCCGTCATTGCCGCTGATGCTGGCCTCTTGCGCGACCTTCTGCTGCATCTGATTGGTAACGCCTTGACCTTCGGAGGACCAGAGCCTCGGATTGCTGTGAGAACCGAACGGCAAGGTGCCGGCTGGAGCATCGCTGTGAGCGATCAGGGTGCGGGGATAGCGCCGGAGTATCAGGAGAGGATCTTCTTGCTCTTCCAGAGACTCGGCTCTCTGGACGAGGCACTGGGCAATGGACTTGGGCTTCCCCTGTGTCGGAAGATCGCCGAACTGCATGGTGGCACACTGACTGTGGAGAGTGCACCTGGCGAAGGAAGCACGTTTAGGTGCTGGCTGCCTGATGCCGGACCACTGCCTGGAGCAGCAGACGCTGGTACCGCCAGCCCCTCTAACCCTGGAGCCTCCAACGGCAGCCTCGAGCGCCCTTCTGACTCCAATGCTGTGTCCCTGGTGATGAGGAGAATCAGAAAGGAGAATGTGGATGCTGGGGAGAGAGCCAAGCAGGCCCTGGCCTTTGAACGCACTGACTTTGACCAAGTCAGATCCCTGATGGAGAACTCTGACAGATGCCAGGACATCAGGAACCTGGCCTTCCTGGGCATTGCCTACAACACCCTGCTGCGCATTGCCGAAATTGCCAGAATCAGAGTGAAGGACATCTCCCGCACCGATGGTGGGAGAATGCTGATCCACATTGGCAGGACCAAGACCCTGGTGTCCACAGCTGGTGTGGAGAAGGCCCTGTCCCTGGGGGTTACCAAGCTGGTGGAGAGATGGATCTCTGTGTCTGGTGTGGCTGATGACCCCAACAACTACCTGTTCTGCCGGGTCAGAAAGAATGGTGTGGCTGCCCCTTCTGCCACCTCCCAACTGTCCACCCGGGCCCTGGAAGGGATCTTTGAGGCCACCCACCGCCTGATCTATGGTGCCAAGGATGACTCTGGGCAGAGATACCTGGCCTGGTCTGGCCACTCTGCCAGAGTGGGTGCTGCCAGGGACATGGCCAGGGCTGGTGTGTCCATCCCTGAAATCATGCAGGCTGGTGGCTGGACCAATGTGAACATTGTGATGAACTACATCAGAAACCTGGACTCTGAGACTGGGGCCATGGTGAGGCTGCTCGAAGATGGGGACtaa

**PACre: CreN(18-59)-nMag**

gccaccATGGCCACTTCCGACGAGGTGAGAAAGAATCTGATGGACATGTTTAGGGATAGGCAGGCCTTCAGCGAGCACACCTGGAAGATGCTGCTGTCTGTGTGCAGATCTTGGGCCGCCTGGTGTAAGCTGAATGGCACCCACACCCTGTACGCCCCCGGGGGCTACGACATCATGGGATACCTGGACCAGATTGGCAACAGGCCAAATCCCCAGGTGGAGCTGGGCCCCGTGGACACCAGCTGCGCTCTGATCCTGTGCGACCTGAAGCAGAAGGACACCCCAATCGTGTATGCCTCCGAAGCCTTTCTGTACATGACCGGCTATTCTAATGCCGAAGTGCTGGGACGGAATTGTCGCTTTCTGCAGAGTCCCGATGGGATGGTGAAGCCCAAGTCTACAAGAAAGTACGTGGACAGCAACACTATTAACACTATTAGAAAGGCCATCGACCGGAACGCCGAGGTGCAGGTCGAGGTGGTGAACTTCAAGAAGAATGGTCAGCGGTTCGTGAACTTCCTGACCATCATCCCCGTGAGGGACGAGACAGGCGAGTACCGGTATTCTATGGGCTTCCAGTGCGAGACCGAGGGCGGCAGCGGGGGAGTGCCCAAGAAGAAGCGGAAGGTGtaa

**PACre: pMag-CreC60**

gccaccATGGTGCCCAAGAAGAAGAGGAAGGTGGGAGGGCACACACTGTACGCCCCAGGCGGTTATGACATCATGGGGTATCTGCGGCAGATCAGAAACAGGCCAAACCCTCAGGTGGAACTGGGCCCCGTGGACACCAGTTGCGCCCTGATTCTGTGTGACCTGAAACAGAAAGACACACCTATCGTGTACGCCTCTGAAGCCTTCCTGTACATGACTGGCTACTCCAATGCCGAGGTGCTTGGCAGAAACTGCAGGTTTCTGCAGAGCCCTGACGGAATGGTGAAGCCCAAATCCACCCGGAAGTACGTGGACTCCAACACCATTAATACCATCAGGAAGGCCATTGATAGAAACGCTGAGGTTCAGGTGGAGGTGGTGAACTTTAAAAAGAATGGCCAGAGGTTCGTGAACTTCCTGACCATCATACCTGTGAGGGACGAGACAGGCGAATACCGGTACTCAATGGGATTTCAGTGCGAGACCGAGGGCACAAACCGGAAGTGGTTTCCTGCCGAGCCCGAGGACGTGAGAGACTATCTGCTGTATCTGCAGGCCCGCGGCCTGGCAGTGAAGACTATCCAGCAGCACCTGGGACAGCTGAACATGCTGCACCGGCGCTCTGGCCTGCCCCGCCCCAGCGACAGCAATGCCGTGAGCCTGGTGATGAGGAGAATCCGGAAAGAAAATGTGGACGCCGGGGAGCGGGCTAAACAGGCTCTGGCCTTCGAGAGGACTGACTTCGACCAGGTGAGGTCTCTGATGGAAAATTCCGACAGGTGTCAGGACATTAGAAATCTTGCCTTCCTGGGAATTGCTTATAATACCCTGCTGCGGATCGCCGAAATCGCCAGGATCAGGGTGAAGGACATTTCCCGCACTGACGGCGGCAGGATGCTGATCCATATCGGCCGGACCAAGACCCTGGTGTCTACCGCTGGCGTGGAGAAAGCTCTGAGCCTCGGGGTGACCAAGCTGGTGGAAAGATGGATCAGTGTGTCAGGCGTGGCTGATGACCCCAACAACTACCTGTTCTGCAGAGTGAGAAAGAATGGCGTGGCCGCCCCCTCCGCTACCAGCCAGCTGAGCACCCGGGCACTGGAGGGAATCTTCGAAGCCACCCACCGCCTGATCTACGGGGCCAAGGATGACAGCGGCCAGAGATACCTGGCCTGGAGCGGCCACTCAGCCCGCGTGGGGGCTGCCCGCGATATGGCTCGGGCAGGCGTGAGCATTCCAGAGATCATGCAGGCCGGCGGCTGGACAAACGTGAACATCGTCATGAACTATATCAGGAATCTGGACAGCGAGACTGGGGCCATGGTGCGGCTGCTGGAGGACGGGGACtga
